# Early molecular changes induced by FLASH irradiation in MCF10A and MDA-MB-231 breast cell lines

**DOI:** 10.3389/fonc.2026.1857611

**Published:** 2026-07-28

**Authors:** Luigi Minafra, Marco Calvaruso, Gaia Pucci, Francesco Sarnari, Giorgio Russo, Valentina Bravatà, Francesco Paolo Cammarata, Fabio Di Martino, Andrea Cavalieri, Laura Bartolomei, Aurora Grassi, Davide Dalfovo, Alessandro Romanel, Marcella Bonanomi, Simonetta Croci, Emanuele Scifoni, Alessandra Bisio, Giusi Irma Forte

**Affiliations:** 1 Institute of Bioimaging and Complex Biological Systems (IBSBC)- National Research Council (CNR), Cefalù, Italy; 2 National Institute for Nuclear Physics, Laboratori Nazionali del Sud, Catania, Italy; 3 Azienda ospedaliero-universitaria pisana (AOUP), U.O. Fisica Sanitaria, Pisa, Italy; 4 National Institute of Nuclear Physics (INFN), Section of Pisa, Pisa, Italy; 5 Center for Instrument Sharing of the University of Pisa (CISUP), University of Pisa, Pisa, Italy; 6 Centro Pisano ricerca e implementazione clinica Flash Radiotherapy (CPFR@CISUP), Presidio S. Chiara, Pisa, Italy; 7 Department of Cellular, Computational and Integrative Biology (CIBIO), University of Trento, Trento, Italy; 8 Institute of Bioimaging and Complex Biological Systems (IBSBC)- National Research Council (CNR), Milan, Italy; 9 Department of Medicine and Surgery, University of Parma, Parma, Italy; 10 Trento Institute for Fundamental Physics and Application (TIFPA), Trento, Italy

**Keywords:** breast cells, FLASH-RT, immunological profiling, mitochondrial genes, transcriptomics

## Abstract

**Introduction:**

We used the non-tumorigenic MCF10A and triple-negative MDA-MB-231 breast cell lines to compare cell response to CONV and FLASH dose-rates under normoxic conditions. Beyond evaluating cell survival and DNA/microtubule damage, we assessed transcriptomic and immunological profiles to describe putative molecular changes. Oxidative stress induced by two different irradiation modalities was also investigated.

**Methods:**

Breast cell lines were irradiated with electron beams at increasing doses of 2, 4, 6, 9, 11, and 15 Gy delivered at either FLASH (230 Gy/s) or CONV (6 Gy/min) dose rates. Survival fractions were determined by clonogenic assay and dose-response curves. DNA damage was quantified by γ-H2AX and 53BP1 foci counting at 0.5, 1, and 24 hours after treatment with 2 and 5 Gy, while microtubule damage was evaluated by confocal microscopy. Transcriptomic profiling was performed by RNA sequencing 24 hours after RT with doses of 9 and 15 Gy. Immunological profiles were analyzed by using Luminex technique at 24, 48, and 72 hours post-RT. The GSH/GSSG ratio was also measured by mass spectrometry at 24 hours post-treatments to assess differences in cellular oxidative status.

**Results:**

Cell survival was comparable between FLASH and CONV regimens in both cell lines. However, a dose-rate effect was observed at the level of early DNA damage, with increased γ-H2AX foci 1 hour after FLASH-RT in both cell lines, and greater persistence of 53BP1 foci at 24 hours in MDA-MB-231 cells, suggesting a dose-dependent response. Immunological profiling showed no qualitative differences between dose rates; nevertheless, MDA-MB-231 cells produced higher levels of several factors after FLASH-RT, whereas MCF10A cells displayed minimal variation. Transcriptomic profiling revealed a broader gene modulation following FLASH-RT, with mitochondrial gene upregulation in MCF10A cells and induction of structural genes in MDA-MB-231 cells. No significant differences in glutathione balance were detected between FLASH and CONV irradiation in either breast cell line.

**Discussion:**

Our findings provide new insights into the early biological responses to ultra-high dose rates *in vitro* under normoxic conditions, suggesting that the dose-rate effect may influence early cellular processes at different levels, without directly affecting cell survival.

## 1 Introduction

New treatment opportunities come from a novel technique, defined as “FLASH Radiotherapy (RT)”, consisting in delivering the prescribed dose at very high dose-rates, at least 100 times greater than those used in conventional regimes. This RT treatment produces the so-called “FLASH effect”, predominantly observed in preclinical experiments, consisting of limited or absent radiation-induced toxicity on healthy tissues, including brain and cognitive preservation, maintaining the tumor control. Such an advantage would allow the delivery of higher doses to the tumor, with limited or no side effects and a consequent widening of the therapeutic window, i.e., maintaining treatment efficacy at the TCP (Tumor Control Probability) level and decreasing the NTCP (Normal Tissue Complication Probability) (1).

The FLASH effect is currently observed at dose levels higher than those used in conventional (CONV) RT, delivered over very short durations (< 200 ms) under ultra-high dose rate (UHDR) conditions using electron or proton beams; conversely, it is reduced at lower doses and with prolonged delivery times (2). However, the relation between the dose/dose rate delivery, the type of target tissue, its oxygenation levels, and, above all, the biochemical and molecular mechanisms underlying FLASH effect still need to be elucidated.

Generally, *in vitro* research offers the possibility to better deepen, at a molecular level, the cellular response to external stimuli. However, the *in vitro* literature collected till now on this topic, failed to unequivocally clarify the FLASH effect mechanisms, only limiting to show a clear dependence by the oxygen levels, as hypoxia (˜O*_2_* < 4%) enhances the sparing effect appearance both in combination with CONV and UHDR irradiation, using doses > 8 Gy of electron or proton beams (3, 4).

As regards the investigation of classical radiobiological endpoints, the current *in vitro* research mainly explored cell survival curves and DNA damage kinetics, searching for differences in the response to the two irradiation modalities (CONV and UHDR). In a recent review, Adrian et al. (5) summarised the *in vitro* data collected from clonogenic assays between 1967 and 2021, displaying non-univocal results and discussing variability dependence on beam delivery parameters and on technical aspects of clonogenic assay preparation. Several other authors investigated the DNA damage pathway, searching for differences in damage entity or kinetics of repair between UHDR and CONV irradiation mode (6–8). In addition to cell survival and DNA damage differences, other biological endpoints have been investigated to study cellular processes differently triggered by UHDR irradiation with respect to CONV. As example, no difference in cell cycle distribution was observed by the group of Adrian et al. (6) in three cell lines subjected to 3 Gy and 6 Gy at 6 and 24 hours post irradiation, while the group of Auer et al. (9) observed a smaller HeLa cell fraction arrested in G2 phase of cell cycle 10 hours post irradiation with 3 Gy by using a pulsed UHDR, respect to continuous and CONV proton SNAKE 20 MeV microbeam. The last authors also showed no difference in cell survival, nor in the micronuclei and apoptosis rates, but reported a slightly lower rate of chromosomal aberration in pulsed irradiated HeLa cells, thus sustaining that the sparing effect is due to less damaged cells under pulsed modality irradiation.

These observations have still not permitted a full comprehension of the biological mechanisms underlying the FLASH effect, which is mandatory in the translation process toward clinical practices.

In this perspective, we used two breast cell lines, the non-tumorigenic MCF10A and the tumorigenic triple-negative MDA-MB-231 breast cancer cells, to explore different responses induced by CONV and FLASH irradiation in normoxic conditions. These two cell lines were previously characterized for their typical response to irradiation by using different types of beams, concerning their own specific cell pathways RT-induced and about the cytokines panel secreted in the culture medium up to 72 hours post-treatment, thus representing optimal *in vitro* models in order to highlight any differences attributable to a dose rate effect (10–16). Also, irradiations were conducted in normoxia, to reveal other dose rate effects not related to hypoxia, as it is an already known FLASH effect favouring factor. In addition to cell survival, DNA and microtubule damage differences, we highlighted cell response in terms of transcriptomic and immunological profiling, and in terms of GSH/GSGG ratio, in order to assess the cellular oxidation response to CONV and FLASH dose rate.

## 2 Materials and methods

### 2.1 Irradiation set-up

All irradiations were carried out at the Centro Pisano FLASH Radiotherapy (CPFR) using the ElectronFlash linear accelerator (17). This device delivers 7 or 9 MeV electron beams and allows modulation of the average dose-rate (ADR) and dose per pulse (Dp) by adjusting the electron beam current and the pulse repetition frequency (PRF).

This flexibility enables maintaining the same Dp and ADR in the FLASH irradiation, varying the dose, and switching from CONV to FLASH irradiation conditions without altering the experimental setup. A summary of all parameters for all the doses of radiation delivered is provided in **Table 1**.

**Table 1:** The table summarizes the radiobiological relevant parameters used in each irradiation condition.

Irradiation modality	Dose per pulse [Gy]	Average dose rate [Gy/sec]	Average dose within a pulse [Gy/sec]	Pulse duration [µs]
FLASH	1	230 Gy/s	210^5^ Gy/s	4
CONV	0,02	6 Gy/min	5000 Gy/s	4

**Figure 1:**
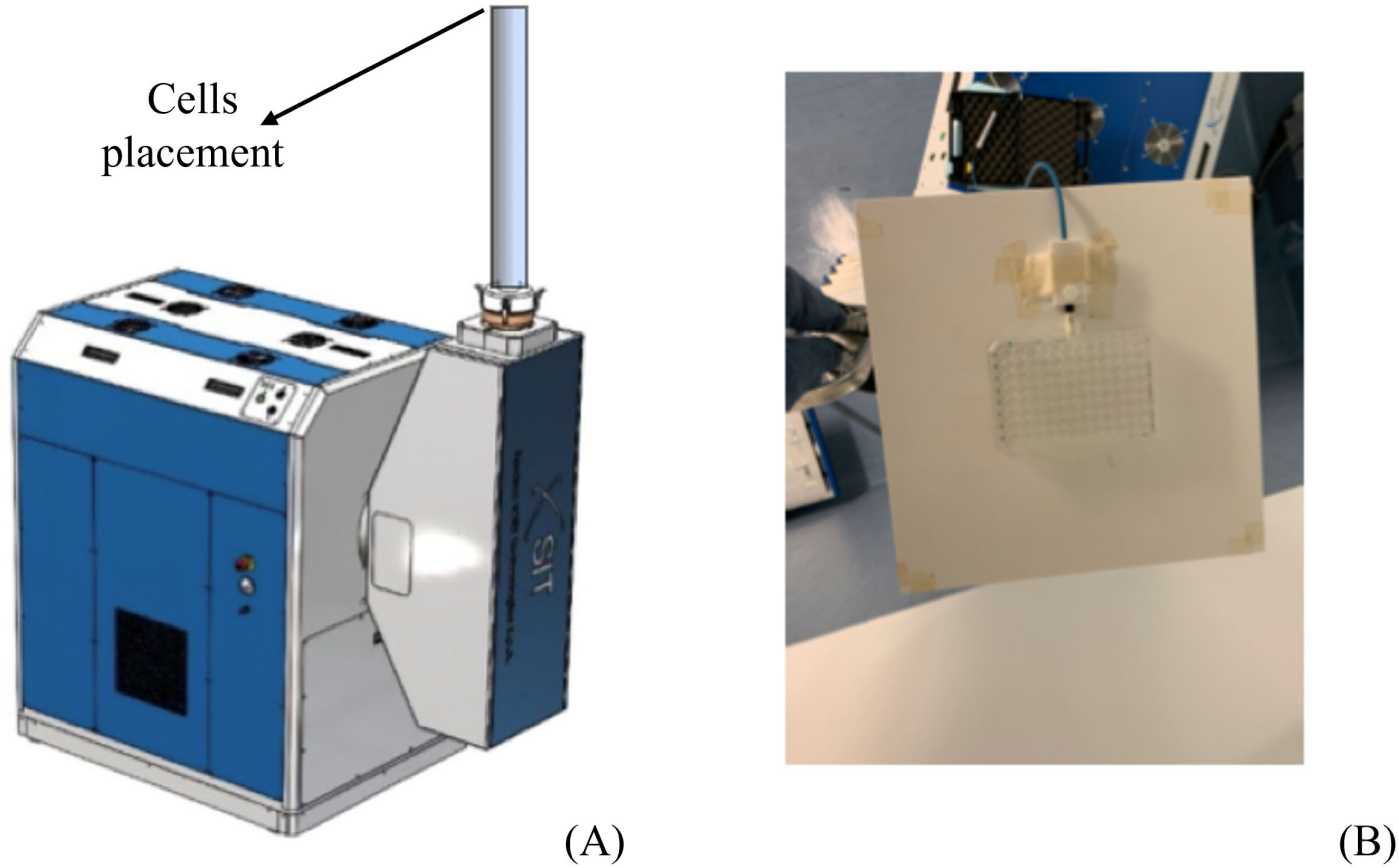
Vertical positioning of the electronFlash device **(A)**. Example of a Petri dish placed on the solid water slab **(B)**.

For all the experiments, both CONV and FLASH irradiations were performed using the 120 mm applicator, ensuring a dose distribution homogeneity within 95% across the entire area of the petri dishes. In this set-up, the linac operated in vertical configuration with the petri dish positioned on the build-up depth, obtained with 12 mm of solid water slab (**Figure 1**).

By varying the PRF, we maintained a constant average dose rate (ADR) of 230 Gy/s across all dose levels used in this study. This value exceeds the 100 Gy/s threshold required to induce the FLASH effect and remains stable regardless of changes in the total delivered dose. Indeed, assuming n pulses and neglecting pulse duration, the ADR is defined as:


$$ADR=\frac{n}{n-1}\;\times DPP\;x\;PRF$$

The dosimetric characterization of our beams was performed using the flashDiamond (fD) detector (18) along with various other dosimeters and measurement techniques, including those developed in-house at CPFR (19, 20) and provided by other centers (21).

The uncertainty of the absolute dose under reference conditions (build-up depth on the beam axis) is within 3%, and the beam uniformity across the irradiation surface used in the experiments, calculated as (Dmax-Dmin)/(Dmax+Dmin)*100%, is within 4%.

The total time of an experimental irradiation session was 2 hours; we continuously monitored the temperature inside the bunker throughout the duration of all experimental sessions and the room temperature throughout the entire period fluctuated between 21 and 22 °C.

The duration of individual irradiations varied from a maximum of 2.5 min (“CONV” mode and dose of 15 Gy) to a minimum of 0.01 s (“Flash” mode and dose of 2 Gy).

### 2.2 Cell cultures, clonogenic assay and survival curves

The human tumorigenic MDA-MB-231 breast cancer cell line and the non-tumorigenic MCF10A breast cell line were purchased from the American Type Culture Collection (ATCC, Manassas, VA, USA) and cultured in standard conditions, as previously reported (22). The passage number range of the cell lines used during irradiation experiments were 19–22 for MCF10A and 10–15 for MDA-MB-231. All experiments were conducted under standard normoxic conditions (21%).

Cell irradiations were performed in CONV and FLASH modality with doses of 2, 4, 6, 9, 11 and 15 Gy. For each dose point, four replicates were used and three independent irradiation experiments were conducted. Clonogenic assay and dose-response curves were carried out as previously described (23).

### 2.3 DNA damage

MCF10A and MDA-MB-231 cells were irradiated in ultra-thin 96 well plate (PhenoPlate-96 TC, Revvity, Milan, Italy), through the slide plate base using the ElectronFlash linear accelerator. Thirty minutes, one hour and 24 hours after irradiation, cells from three independent biological replicates were fixed in 4% formaldehyde (Sigma-Aldrich, Milan, Italy) for 15 min at room temperature (RT) After fixation, cells were blocked in 3% BSA-0.3% Triton X-100 PBS solution for 45 min at RT. Cells were then incubated for 1 h at RT with the following primary antibodies diluted in 3% BSA-PBS: Anti-phosphorylated H2AX (clone 9F3, Abcam, Prodotti Gianni, Milan, Italy) and anti-53BP1 (ab21083, Abcam). Subsequently, cells were washed once with PBS and incubated for 1 h at RT with Alexa Fluor 488- and Alexa Fluor 594-conjugated secondary antibodies diluted in 3% BSA-PBS. Nuclei were counterstained using Hoechst 33342 (Thermo Fisher Scientific), diluted 1:2000 in PBS. Fluorescence images were acquired using the ImageXpress micro-confocal high-content imaging system (Molecular Devices, San Josè, CA, USA) at CIBIO HTS (High Throughput Screening) facility. Images were captured from 6 fields of view (FOV) per well using a 20× Plan Apo Lambda objective (0.75 NA) in spinning disk confocal mode. For each FOV, a Z-stack of 4 focal planes with 3µm steps was acquired. A Maximum Intensity Projection (MIP) was subsequently generated from each image set and utilized for all downstream quantitative analyses. Filter sets were configured as follow: Ex. 377/54 nm - Em. 432/36 nm for Hoechst signal; Ex. 475/28 nm - Em. 536/40 nm for Alexa Fluor 488; Ex. 576/23 nm - Em. 624/40 nm for Alexa Fluor 594.Image Analysis was performed using MetaXpress software (version 6.7.2.290; Molecular Devices, San Josè, CA, USA). Nuclei were identified by segmenting the Hoechst channel. For each identified nucleus, DNA damage foci were quantified in both the Alexa Fluor 488 and Alexa Fluor 594 channels by applying specific size (dimension) and intensity thresholds. An average of 1500 nuclei were assessed per experimental condition, and the mean number of DNA damage foci per nucleus was aggregated and calculated for each well.

### 2.4 Microtubule damage by immunofluorescence for confocal microscopy

Four and a half hours after irradiation, the cells from two technical replicates of two independent irradiation experiments, were pre-fixed using a PHEM 1x buffer supplemented with 0.2% glutaraldehyde and 0.25% Triton X-100. Fixation was carried out in a PHEM 1x buffer containing 4% PFA and 0.2% glutaraldehyde. Aldehyde reduction was achieved by treating the samples with freshly prepared sodium borohydride (NaBH_4_, 1 mg/mL in PBS). The MDA-MB-231 cells were labelled with mouse monoclonal anti-α-tubulin antibody diluted at 1:400, following the manufacturer’s instructions (Merck KGaA). Then, cells were stained with goat anti-mouse Alexa Fluor 647 (Invitrogen, Thermofisher) diluted at 1:400. Then, cells were fixed in 3,7% PFA, incubated in NH_4_Cl solution and stored at 4 °C. The acquisition for confocal laser scanning microscopy (CLSM) was performed using the same acquisition parameters (px dimension (nm); px Dwell time (ms); distance between slice Dz (mm), gain and laser intensity) throughout all observations, to obtain consistent image stacks suitable for a quantitative data analysis. Image analysis was performed by using the Fiji/ImageJ software (NIH, USA). Since the height of the cellular monolayer may vary from field to field, the number of slices in each stack may therefore not be the same. For each image stack, an intensity selection map was created by Z- projecting the maximum value over the x,y plane.

For every intensity selection map, the analysis was performed by segmenting the image of the selection map using CELLPOSE software (24), a procedure that generates a corresponding ROI for each of the detected cells. Following this segmentation, in order to assess the stability and robustness of the quantitative measurements with respect to the definition of the ROI boundaries, additional ROIs were generated through controlled margin contraction operations (10 px). This is mainly to remove the cell boundaries where filament accumulation occurs, resulting in signal saturation. The Median of the Gray-level intensity Value within each cell (ROI) was calculated. Subsequently, for each group (CTRL, irradiated 4 Gy CONV, irradiated 4 Gy FLASH, irradiated 6 Gy CONV, irradiated 6 Gy FLASH, irradiated 9 Gy CONV, irradiated 9 Gy FLASH), the Mean Gray-level intensity. Values of the distribution were standardised against the control group (% MGV) and analysed.

### 2.5 RNA extraction, retrotranscription, qRT-PCR

RNA extractions were performed from MCF10 and MDA-MB-231 cells, 24 hours post irradiation with the doses of 9 and 15 Gy delivered in CONV or FLASH modality, by using TRIzol Plus RNA Purification Kit, according to the manufacturer’s protocol (Thermo Fisher SCIENTIFIC, Waltham, Massachusetts, USA). cDNA synthesis and qRT-PCR were performed as previously reported (25).

In order to validate the gene signature obtained by RNA sequencing and bioinformatic analysis, some randomly selected among the most significant up-regulated genes in the FLASH *vs* CONV comparison were assayed for each dose/cell line. Also, upregulation of common mitochondrial genes observed in response to the doses of 9 Gy and 15 Gy (9∩15 Gy) FLASH-treated MCF10A cells was validated, including negative controls containing no retrotranscribed template, in order to exclude that amplified products could derive from mitochondrial DNA amplification. A list of used primers is available in **Supplementary File 1**.

### 2.6 RNA- sequencing and bioinformatic analysis

RNA-seq libraries were prepared from total RNA extracted 24 hours post irradiation, from MDA-MB-231 and MCF10A cells, untreated and treated with 9 and 15 Gy delivered in CONV or FLASH modality. For each condition, we analyzed two independent biological replicates. Libraries’ preparation and sequencing were performed by the CIBIO Next Generation Sequencing facility using the CORALL RNA-Seq V2 Library Prep Kit (Lexogen, Vienna, Austria). Ribo-depleted RNAs serve as templates for CORALL reverse transcription, and no prior RNA fragmentation is required. Reactions were performed following the manufacturer’s instructions. Purification steps were based on the use of the Ampure XP magnetic beads (Beckman Coulter, Milan, Italy). After quantification with Qubit (Thermo Fisher Scientific, Milan, Italy), and quality control using the LabChip GX (PerkinElmer, Waltham, MA, USA), the indexed libraries were pooled, quantified by qPCR using the KAPA Library Quantification Kit (Roche, Basel, Switzerland), and sequenced on the NovaSeq 6000 Sequencing System (Illumina, San Diego, CA, USA). portal, which integrates Gene Ontology (GO) terms, pathway databases (e.g., KEGG, Reactome), and other functional annotations to identify significantly over-represented biological processes and molecular pathways within the DEG lists.For each sample, raw sequencing data were pre-processed to extract the 10-base pair UMI sequence from each read and append it to the read header for subsequent duplicate identification. Following UMI extraction, Cutadapt v4.1 (26) was employed to remove adapter sequences and trim low-quality bases of the reads to ensure high-quality data for alignment. The pre-processed reads were aligned to the human reference genome (assembly version GRCh38) using the aligner STAR v2.7.10b (27). After alignment, PCR duplicates were identified and removed using UMI-tools v1.1.4 (28), which collapses reads with the same alignment coordinates and identical UMI into a single count, thus mitigating amplification bias. The resulting de-duplicated alignment files were stored and indexed in BAM format. Gene-level expression was quantified by counting the number of reads mapping to each gene annotated in the reference genome. This was performed using HTseq v2.0.2 (29). Throughout the workflow, overall quality control metrics from all steps were aggregated and summarized into a single comprehensive report using MultiQC (30) to ensure data integrity and consistency across all samples. Differential Gene Expression (DEG) analysis was conducted to identify genes with significant expression changes between experimental conditions using DESeq2 package (31). The main comparisons included: (i) irradiated (both 9 Gy and 15 Gy) versus untreated controls within each cell line (MDA and MCF); (ii) FLASH versus CONV RT at the same dose within each cell line; and (iii) the response to each treatment between the two cell lines (MDA vs. MCF). Genes were considered differentially expressed based on statistical significance (adjusted *p-value* < 0,05) and the magnitude of change (absolute log_2_ fold change >1). To understand the biological implications of the observed gene expression changes, functional enrichment analysis was performed on the lists of up- and down-regulated genes. This analysis was conducted using the Metascape (32) web.

### 2.7 GSH/GSSG quantification

Cell pellets from two technical replicates of two independent irradiation experiments, were extracted using an ice-cold acetonitrile–water solution (70:30, v/v). Samples were incubated at 80 °C for 10 min and subsequently sonicated twice for 5 second at 70% power. Following centrifugation at 12,000 × g for 10 min, the supernatant (aqueous phase) was transferred to glass inserts and dried using a centrifugal vacuum concentrator (Concentrator plus/Vacufuge plus, Eppendorf, Hamburg, Germany) at 30 °C for approximately 2,5 hours. Dried extracts were reconstituted in 100 μL of acetonitrile–water (70:30, v/v) prior to analysis. Metabolite analysis was performed using an ultra-high-performance liquid chromatography–triple quadrupole mass spectrometry (UHPLC–TQ-MS/MS) platform. Chromatographic separation was carried out on an Agilent 1290 Infinity II Bio UHPLC system equipped with an Agilent InfinityLab Poroshell 120 HILIC-Z column (2.1 × 100 mm, 2.7 μm particle size) (Agilent Technologies, Santa Clara, USA). The column temperature was maintained at 25 °C, and 10 μL of sample was injected for each analysis. The mobile phases consisted of 10 mM ammonium acetate in water (pH 9) supplemented with 2,5 μM InfinityLab deactivator additive (Agilent Technologies) (mobile phase A) and 10 mM ammonium acetate in acetonitrile: water (85:15, v/v; pH 9) (mobile phase B). The chromatographic gradient was as follows: 0 min: 5% A; 2 min: 5% A; 12 min: 50% A; 13 min: 5% A; 18 min: 5% A. The flow rate was set to 0,25 mL/min.

Mass spectrometric detection was performed using an Agilent 6495D TQ mass spectrometer equipped with an electrospray ionization (ESI) Jet Stream source operating in negative ion mode. Source parameters were as follows: gas temperature 200 °C, gas flow 13 L/min, nebulizer pressure 35 psig, sheath gas temperature 250 °C, sheath gas flow 11 L/min, capillary voltage (VCap) 2600 V, fragmentor voltage 166 V, and nozzle voltage 1700 V.

Multiple reaction monitoring (MRM) transitions for reduced glutathione (GSH) and oxidized glutathione (GSSG) were optimized using the MRM optimization tool in Agilent OpenLab Control Panel acquisition software (version 3.6). The monitored transitions, collision energies, and retention times were reported in **Table 2**. Data processing and quantification were performed using Agilent MassHunter Quantitative Analysis for QQQ (version 12.1). The first MRM transition was used for quantification and the second for qualification. Absolute quantification was achieved using external standard calibration curves. Metabolite concentrations were normalized to protein content, determined by the Bradford assay and expressed as mg of total protein.

**Table 2:** GSH and GSSG transitions, collision energies, and retention times.

Analytes	Precursor m/z (M-H)-	Product m/z	CE (V)	RT (min)
GSH	306,1	127,9	19	5,55
		142,8		
GSSG	611.2	271,9	26	7,5
		305,8		

### 2.8 Immunological profiling

The immunological profiles of untreated and treated MCF10 and MDA-MB-231 cells with the doses of 9 and 15 Gy, delivered by using CONV and FLASH dose-rate, were analysed at the time points of 24, 48 and 72 hours post irradiation. In detail, cells were seeded 24 hours before irradiation and twin plates of untreated samples were prepared with the same number of cells for each time point/dose/dose rate to be tested. At the established time points, the irradiated and untreated conditioned mediums (CM) were collected and processed as previously described (11, 14). Then, samples were tested for a panel of 20 cytokines, chemokines and growth factors (Interleukin IL-1α, IL-1β, IL-2, IL-4, IL-5, IL-6, IL-7, IL-8, IL-10, IL-12p70, IL-13, IL-17A, IFN-γ, MCP-1, MIP-1α, GM-CSF, G-CSF, TGF-β1, TGF-β2, TGF-β3), using Bio-plex kit according manufacturer’s instructions (BioRad, Milan, Italy). Samples were analysed on a Luminex 100 device and data were analysed using the Bio-Plex Manager software (BioRad, Munich, Germany). The concentration of each molecule, expressed in pg/ml, was obtained as the mean of quadruplicate measurements (two technical replicates from two independent irradiation experiments), from data interpolation with eight-point standard curves created for each protein.

### 2.9 Statistics

Data reported in **Tables 3** and **4** represent normalised expression levels of cytokines, chemokines and growth factors for each treated sample versus untreated ones. The unpaired *t-*test with Welch correction was applied on two-group comparisons, to test if the means of cytokine concentration of CONV and FLASH irradiated samples were significantly different at 72 hours post- irradiation with 9 Gy or 15 Gy. In addition, second-order polynomial curves were obtained by data fitting in order to describe the cytokine kinetics of production during the 24, 48 and 72 hour time points, by using MATLAB (The MathWorks, Inc., United States) and a *t*-test was applied to search for significantly different CONV and FLASH kinetics curves. Differences were considered significant when a *p-*value < 0,05 was obtained.

**Table 3:** Normalized quantities of 20 cytokines, chemokines and growth factors in treated MCF10A cell lines with respect to untreated controls at time point 0.

Analyte groups	Analytes	Irradiation modality	24 h			48 h			72 h		
			2 Gy	9 Gy	15 Gy	2 Gy	9 Gy	15 Gy	2 Gy	9 Gy	15 Gy
Pro-inflammatory cytokines	IL-1α	CONV	1,03	1,16	1,18	1,26	1,38	1,45	1,50	2,42	3,08
		FLASH	1,16	0,77	1,18	1,28	1,45	1,52	1,63	2,36	3,18
	IL-6	CONV	1,14	1,22	1,60	1,39	1,32	1,59	1,34	1,44	1,85
		FLASH	1,18	0,88	1,55	1,29	1,36	1,64	1,37	1,34	1,80
Chemokines	IL-8	CONV	0,90	0,81	0,99	1,81	1,24	1,15	2,74	1,87	1,69
		FLASH	0,95	0,67	1,03	1,68	1,26	1,16	2,75	1,77	1,67
	MIP1- α	CONV	0,94	1,04	1,03	1,07	1,04	1,08	1,21	1,11	0,96
		FLASH	1,36	0,82	0,89	1,18	1,13	0,92	1,33	1,97	1,41
Immuno-modulatory cytokines	IL-2	CONV	0,92	0,81	1,00	1,27	1,06	1,02	1,69	1,44	1,29
		FLASH	1,04	0,69	1,06	1,29	1,19	0,85	1,71	1,31	1,31
	G-CSF	CONV	1,02	0,97	1,32	2,25	1,84	2,08	3,22	2,81	2,84
		FLASH	1,04	0,80	1,27	2,02	1,94	2,06	3,20	2,60	2,80
	GM-CSF	CONV	0,98	0,90	0,99	1,51	1,34	1,32	1,69	1,86	1,98
		FLASH	0,80	0,40	1,12	1,25	1,30	1,15	1,61	1,74	1,98
TGF- β family	TGF- β1	CONV	0,94	0,53	0,00	1,06	0,54	0,55	1,13	1,13	1,12
		FLASH	0,98	0,91	1,03	1,13	1,01	1,14	1,24	1,20	1,22
	TGF- β2	CONV	0,92	0,60	0,00	2,27	0,68	1,54	3,77	3,03	2,43
		FLASH	0,99	0,83	0,93	1,91	1,42	1,32	4,04	2,99	2,33
	TGF- β3	CONV	0,96	0,69	0,06	1,04	0,51	0,60	1,28	0,96	0,97
		FLASH	0,85	0,68	0,81	1,04	0,87	0,83	0,99	0,95	0,73

Values, expressed as mean of quadruplicated (in term of pg/ml), were normalized using matched values from CM of untreated MCF10A cells. Significativity was evaluated by means of Unpaired t test with Welch correction (*p<0,05).

**Table 4:** Normalized quantities of 20 cytokines, chemokines and growth factors in treated MDA-MB-231 cell lines with respect to untreated controls at time point.

Analyte groups	Analytes	Irradiation modality	24 h			48 h			72 h		
			2 Gy	9 Gy	15 Gy	2 Gy	9 Gy	15 Gy	2 Gy	9 Gy	15 Gy
Pro-inflammatory cytokines	IL-1α	CONV	0,00	0,00	0,00	0,84	0,00	0,84	8,57	17,27	13,40*
		FLASH	0,00	0,00	0,00	2,74	0,00	0,84	6,60	18,42	15,50*
	IL-1β	CONV	0,00	0,00	0,00	0,00	0,00	0,78	3,40	10,09*	9,39
		FLASH	0,00	0,00	0,00	4,22	0,78	0,00	0,78	14,15*	12,78
	IL-6	CONV	1,20	1,10	1,20	3,02	4,03	2,41	19,13	52,43*	18,47*
		FLASH	1,40	1,30	1,30	3,40	4,92	3,10	18,60	64,60*	27,80*
Chemokines	IL-8	CONV	1,13	1,06	1,36	1,77	2,04	2,29	8,06	0,00	13,00
		FLASH	1,00	1,00	1,30	1,94	2,13	2,21	8,61	0,00	14,50
	MCP-1	CONV	1,14	1,05	1,23	2,17	2,15	2,35	3,05	4,74	3,45*
		FLASH	1,16	1,10	1,32	2,08	2,42	2,54	3,24	4,53	4,18*
	MIP1-α	CONV	1,00	0,83	1,21	1,30	1,17	1,26	1,75	1,98*	1,92
		FLASH	1,24	1,05	1,22	1,42	1,34	1,40	1,67	2,14*	2,03
Immuno-modulatory cytokines	IL-4	CONV	1,47	1,47	1,47	2,06	2,06	1,87	2,85	3,14	3,00
		FLASH	1,58	1,68	1,87	2,83	2,39	2,53	2,70	3,41	3,00
	IL-7	CONV	0,00	0,00	0,00	0,00	0,00	0,00	4,10	5,54	6,86
		FLASH	0,00	0,00	0,00	0,00	0,00	1,01	5,54	5,41	6,86
	IL-2	CONV	1,17	1,21	1,48	1,76	1,76	2,04	4,12	5,90	4,64*
		FLASH	1,24	1,21	1,55	1,76	1,96	2,11	3,74	5,76	5,13*
	G-CSF	CONV	0,00	0,00	0,00	0,00	0,00	0,00	6,55	26,98*	16,40*
		FLASH	0,00	0,00	0,00	7,39	0,00	0,00	4,73	30,08*	21,05*
	GM-CSF	CONV	1,03	1,00	1,49	1,52	1,86	2,50	4,42	9,18	9,63
		FLASH	1,00	1,00	1,45	1,78	1,77	2,30	4,53	9,50	9,25
TGF- β family	TGF-β1	CONV	0,97	1,07	0,99	1,09	1,29	1,32	1,53	1,51	1,87
		FLASH	0,90	1,02	1,10	1,31	1,39	1,57	2,09	1,57	1,91
	TGF-β2	CONV	0,90	0,90	0,86	1,57	1,32	1,22	2,93	1,93	1,87
		FLASH	0,85	0,93	0,89	1,86	1,27	1,21	3,49	1,91	1,54
	TGF-β3	CONV	1,09	1,09	0,96	1,53	1,64	1,43	1,43	1,72	1,83
		FLASH	1,02	0,94	0,9	1,52	1,29	1,51	1,86	1,13	1,17

Values, expressed as mean of quadruplicated (in term of pg/ml), were normalized using matched values from CM of untreated MDA-MB-231 cells. Significativity was evaluated by means of Unpaired t test with Welch correction (*p<0,05).

**Figure 2:**
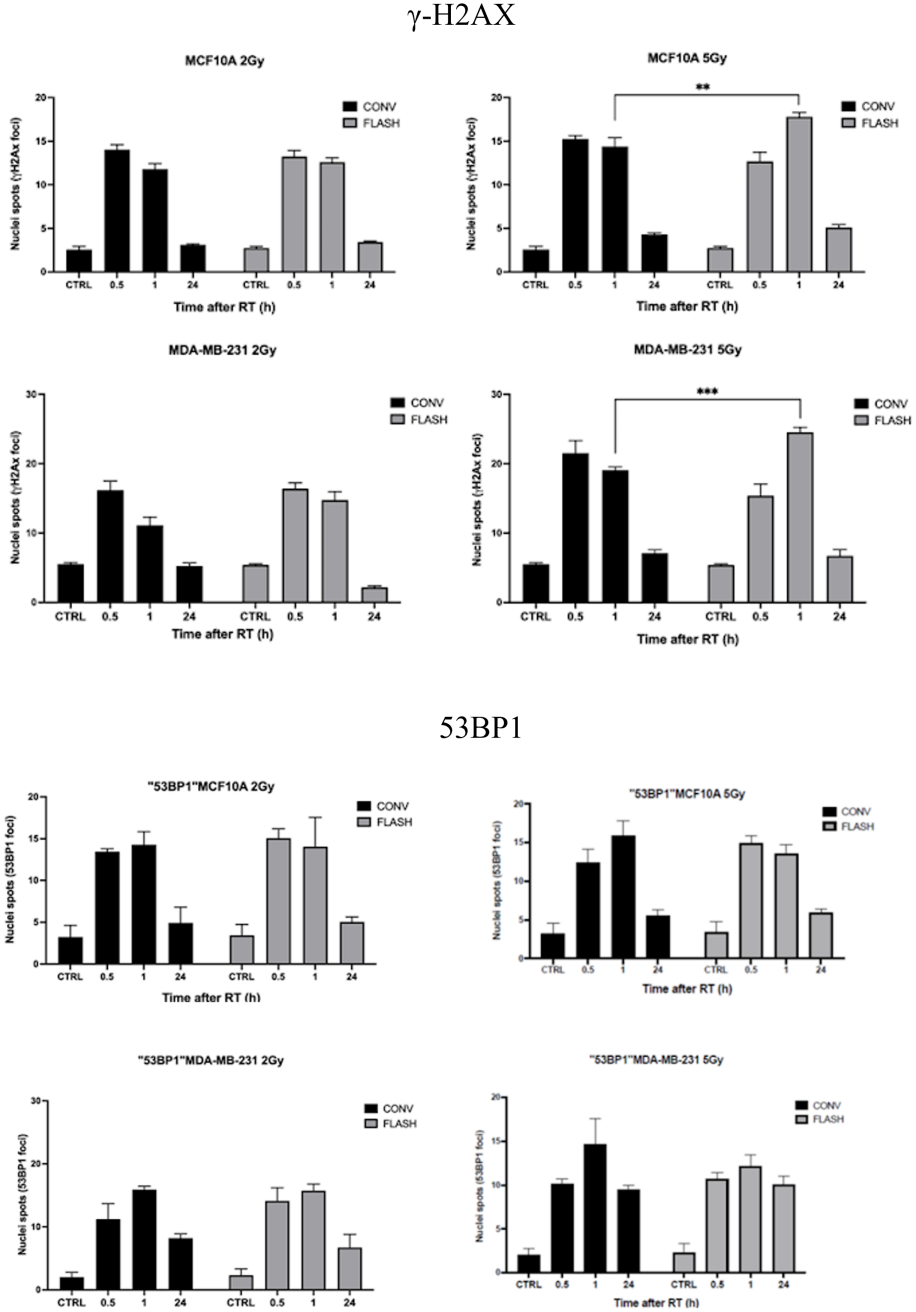
γ-H2AX and 53BP1 foci count in MCF10 A and MDA-MB-231 cell lines treated with the doses of 2 and 5 Gy and CONV- and FLASH- RT dose rates. The analysis was conducted 0.5, 1 and 24 h post irradiation. **p<0.01; ***p<0.001.

The data shown in **Figure 2** are the averages and standard deviations of at least three independent biological replicates and statistical significance was calculated using the unpaired Student’s t test.

As regards the microtubule damage comparison, pairs of groups were compared using the Mann-Whitney test with Bonferroni correction for multiple testing.

For RNA-seq data, statistical analysis of differential gene expression was performed using DESeq2 on raw gene-level count data. Counts were normalized using size factors to account for differences in library size across samples, and gene expression was modeled using a negative binomial generalized linear model. For each predefined contrast, statistical significance was assessed using Wald tests, and *p*-values were adjusted for multiple testing using the Benjamini-Hochberg false discovery rate procedure. Functional enrichment analysis was performed with Metascape, where predefined biological processes and pathway terms were tested for over-representation among the differentially expressed genes relative to a suitable background gene set, with adjustment for multiple testing of enriched categories.

## 3 Results

### 3.1 Cell survival curves

**Figure 3:**
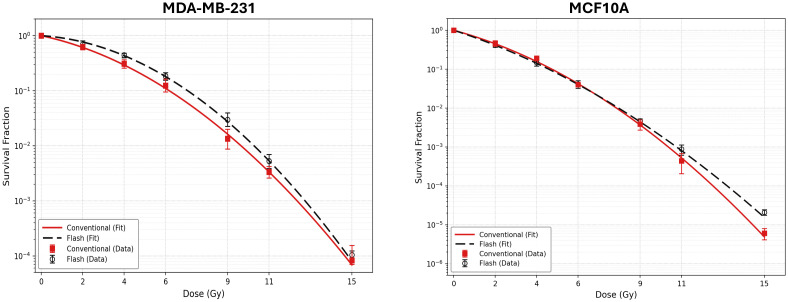
Dose response curves of MDA-MB-231 and MCF10A cell lines irradiated with CONV (red curves) and FLASH (black curves) dose-rates.

To evaluate the effects of CONV and FLASH irradiation on cell survival, non-tumorigenic MCF10A and the triple negative breast cancer (TNBC) MDA-MB-231 cell lines were exposed to electron beams with increasing doses of 2, 4, 6, 9, 11 and 15 Gy and two different dose rates: 230 Gy/sec for FLASH and 6 Gy/min for CONV. The survival fraction (SF) was measured for both irradiation modalities by means of clonogenic assay, to determine whether the FLASH regimen induced a different response compared to CONV treatments (**Figure 3**). In both cell lines, no significant differences were observed between the two irradiation conditions for each of the doses tested. For the highest doses from 9 to 15 Gy, the SF values ​​were very low; these results were probably due to a related dose effect, which prevented the selection of radioresistant clonogens. In the non-tumorigenic MCF10A cells, SF decreased in a dose-dependent manner, and the response to FLASH irradiation was comparable to that of the CONV regimen. Also in the tumorigenic MDA-MB-231 cells, the survival curves for FLASH and CONV irradiation were nearly similar, indicating that the different dose rates did not exert significant effects on the viability of either cell line under the experimental conditions used.

### 3.2 DNA damage

The DNA damage analysis was performed on the two cell lines by means of γ-H2AX and 53BP1 foci count at 0,5, 1 and 24 hours post treatment with the doses of 2 and 5 Gy and the CONV- and FLASH-RT dose rates. **Figure 2** reports the kinetics of appearance of both the γ-H2AX and 53BP1 foci in samples irradiated with the two different dose rates. A significant increase of γ-H2AX foci is observable 1 hour post-treatment with 5 Gy of FLASH-RT in both the cell lines, compared to CONV irradiation (dose-rate effect). This difference disappeared at 24 hours and had no corresponding increase in 53BP1 foci 1 hour post-treatment. Furthermore, a greater persistence of double-strand break damage (53BP1) is observable at 24 hours post-treatment in the MDA-MB-231 cell line with the dose of 5 Gy using both the dose rates (dose effect).

### 3.3 Microtubule damage in MDA-MB-231 cells

**Figure 4:**
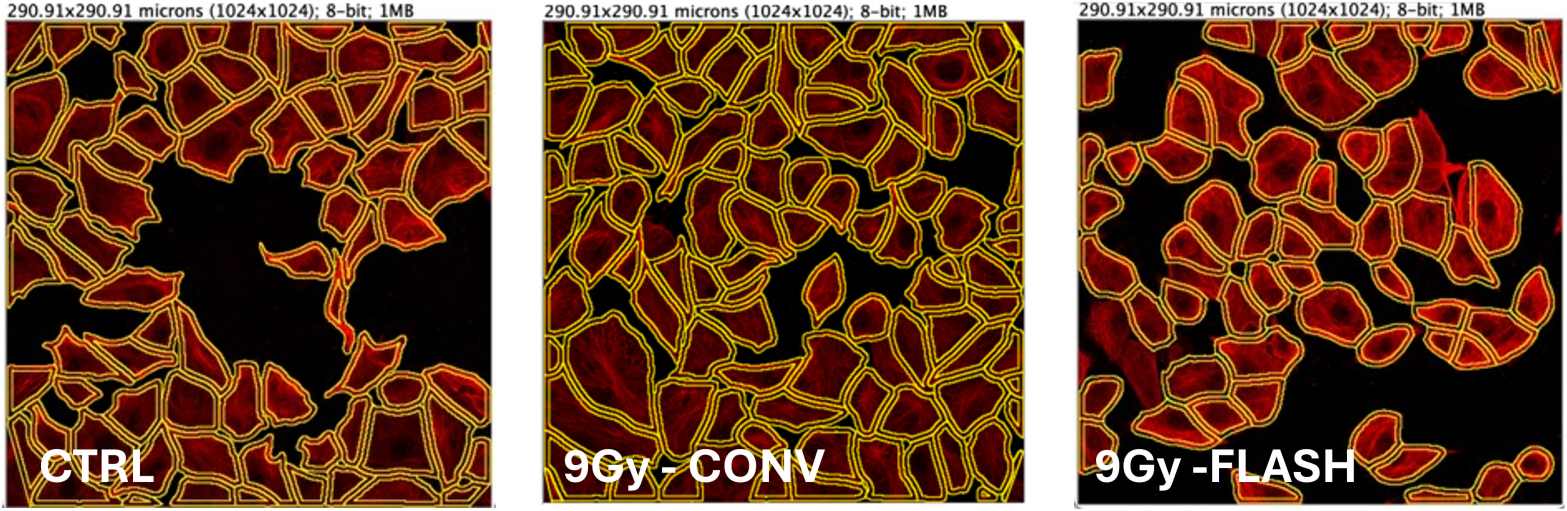
Confocal α−tubulin (Alexa Fluor 647) images of MDA-MB-231 cells: Control, 9 Gy Conv, and 9 Gy FLASH irradiation. The intensity projection along the z-axis onto the x,y plane is shown, with yellow lines outlining ROIs obtained with CELLPOSE software and the final ROIs after contraction operations (10 px).

**Figure 5:**
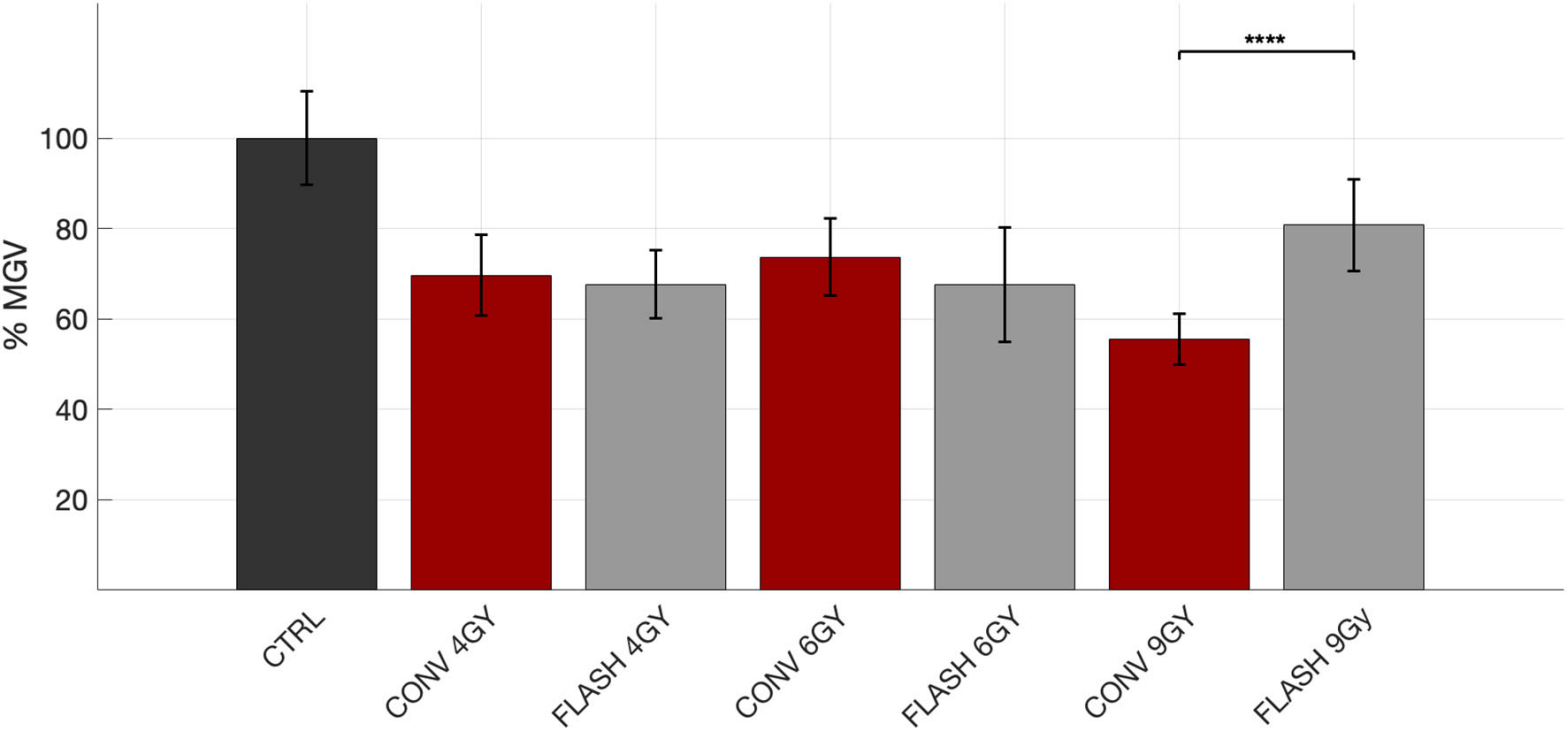
Comparison of the percentage MGV in MDA-MB-231 cells irradiated with electrons in FLASH and CONV modes at doses up to 9 Gy. Error bars represent the interquartile range (IQR). No significant differences were observed between CONV and FLASH irradiation at 4 Gy and 6 Gy. In contrast, at 9 Gy, FLASH irradiation resulted in significantly higher %MGV values compared with CONV irradiation (****p<0.0001), as indicated by the significance bar and asterisks.

The damage to microtubules (MTs) was investigated by confocal microscopy. ROIs were identified as described in the Materials and Methods and an example is shown in **Figure 4**. Subsequently, the Median of Gray-level intensity Values (MGV) was calculated within the ROIs identified in irradiated samples and normalised with respect to the corresponding controls (**Figure 5**). The data presented in **Figure 5** show that, in MDA-MB-231 cells irradiated with electrons in CONV mode, the Median Grey-level intensity value (% MGV) decreases, with the reduction becoming progressively more pronounced as the dose increases up to 9 Gy, indicating a defective labelling due to microtubule damage induced by irradiations. For cells irradiated in FLASH mode, doses of 4 Gy and 6 Gy result in an overall decrease in % MGV similar to that observed with the CONV mode, with no significant differences. However, at 9 Gy, whilst cells treated in CONV mode continue to show a significant (p < 0,001) reduction in % MGV, those treated in FLASH mode show a reversal of this trend, with a lower significant reduction compared to the CONV sample (p < 0,001). These results confirm MTs as a target of ionising radiation damage, highlight a dose-dependent trend in CONV mode up to 9 Gy, indicating a differential response between CONV and FLASH that will require further investigation.

### 3.4 Gene expression profiling

**Figure 6:**
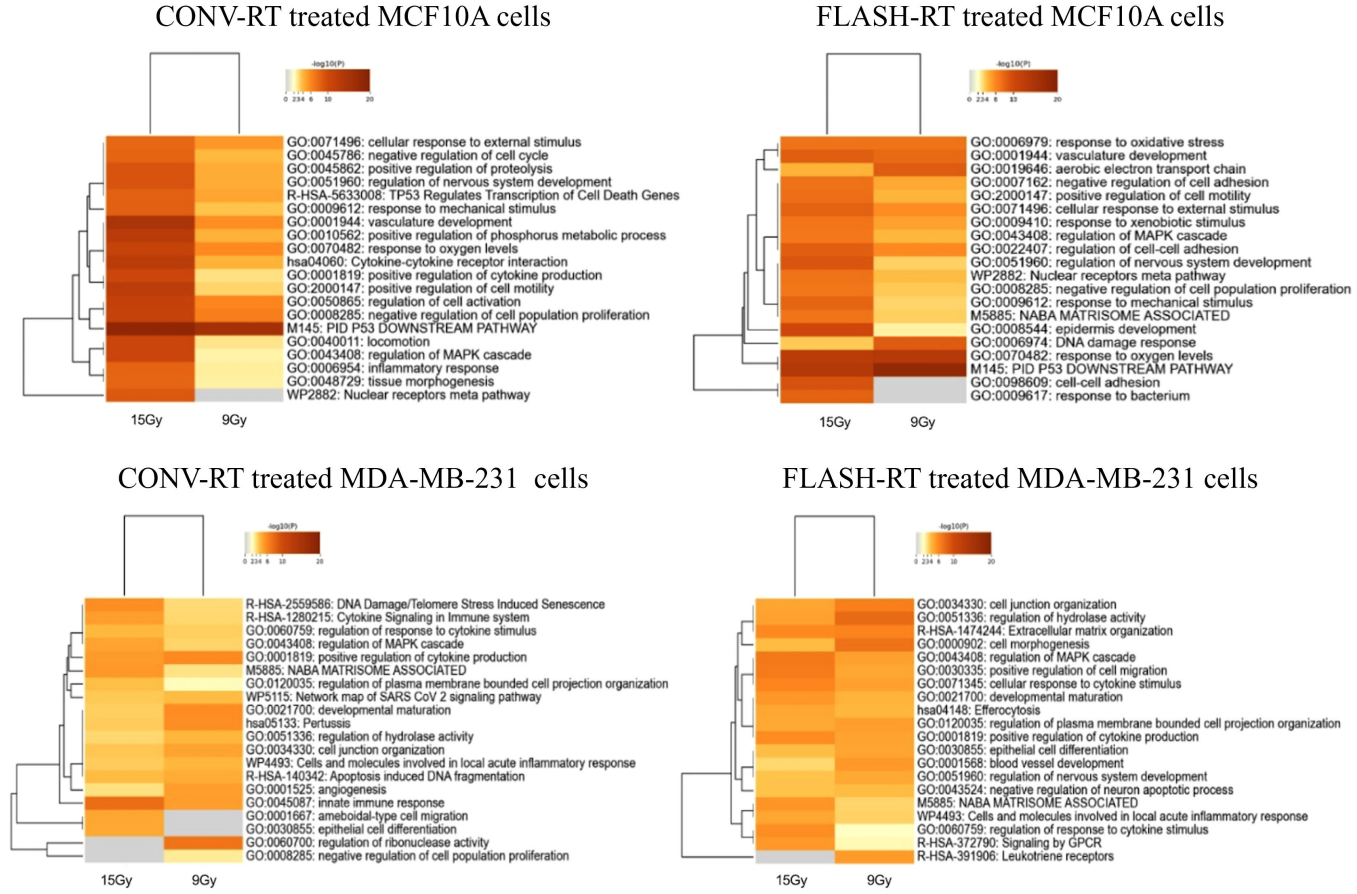
Heatmaps showing common deregulated pathways in response to the doses of 9 Gy and 15 Gy (9∩15) for each type of irradiation mode, CONV-RT and FLASH-RT.

Differential gene expression profiling was investigated by using an RNA-sequencing approach on total RNA extracted from untreated and treated MCF10A and MDA-MB-231 cells 24 hours post irradiation with the doses of 9 and 15 Gy, delivered with CONV (6 Gy/min) or FLASH irradiation (230 Gy/s). RNA sequencing data have been deposited in the BioProject database under the accession number PRJNA1493858. Firstly, the bioinformatic analysis was applied to compare the molecular profiling between the *treated_ vs_untreated* configurations for each dose (9 and 15 Gy), dose rate (CONV and FLASH) and for both cell lines studied. Numbers of significantly down- and up- regulated genes are reported for each comparison analyzed (**Table 5**). Notably, FLASH irradiation was able to deregulate a higher number of genes in both cell lines, especially with the higher dose of 15 Gy (**Table 5**). In addition, heatmaps were created to search for significant common deregulated pathways in response to the doses of 9 Gy and 15 Gy for each type of irradiation modality, CONV- and FLASH-RT (**Figure 6**). This analysis revealed that the dose rate modifies the lists of significant pathways commonly activated by 9 and 15 Gy (9∩15) in both the two cell lines. Indeed, few pathways perfectly matched among the 9∩15 Gy_CONV and the 9∩15 Gy_FLASH lists (“*Naba Matrisome Associated*” and “*negative regulation of cell proliferation*” in MCF10A; “*regulation of nervous system development*”, “*positive regulation of cytokine production*” and “*regulation of MAPK cascade*” in MDA-MB-231 cells). Other differently activated pathways in the MCF10A_CONV sample are related to damage, apoptosis, inflammation, development and differentiation, whereas in the MCF10A_FLASH sample, the response is related to oxidative stress, mechanical stimuli, adhesion and motility, and p53 downstream pathways. Similarly, in the MDA-MB-231_CONV sample, the activated signalling concerns the response to external and mechanical stimulus, to oxygen levels, inflammation and p53 downstream pathways, while in the MDA-MB-231_FLASH sample, they are related to regulation of cell junction, blood vessel and extracellular matrix organisation, inflammation, motility and migration. Thus, in order to better highlight the differences in molecular profiling obtained following cell treatments with the two different dose-rates, a direct comparison between deregulated gene lists in *FLASH vs CONV* configurations was carried out for each dose of 9 and 15 Gy and in both cell lines. The direct comparison revealed very few significant up-regulated genes by FLASH-RT treatment with respect to the CONV dose rate (**Table 5**), except for 15 Gy *FLASH vs CONV.* MDA-MB-231 cells in which no difference was reported. **Tables 6** and **7** reports these few up-regulated genes. Interestingly, no quantitative, but qualitative differences were revealed in the molecular profile induced by FLASH-RT in both cell lines. Indeed, in MCF10A cells, the molecular response to FLASH-RT was characterized by the specific activation of numerous genes belonging to the mitochondrial respiratory chain, both in 9 and 15 Gy treated samples. On the other hand, structural and signal transduction genes featured the molecular response to 9 Gy of FLASH irradiation in MDA-MB-231 cells. In order to validate the gene signature featuring MDA-MB-231–9 Gy and MCF10A 9∩15 Gy configurations, some randomly selected among the most significant up-regulated genes were also assayed by qRT-PCR. In this regard, all the genes tested were validated and these are reported in **Tables 6** and **7**. In addition, upregulation of mitochondrial gene expression was validated, including no retrotranscribed template, in order to exclude the presence of mitochondrial DNA template, as a negative control.

**Table 5:** Number of deregulated genes in response to 9 and 15 Gy FLASH- or CONV-RT treatment with respect to untreated MCF10 and MDA-MB-231 cells.

Treated vs Untreated comparisons			
MCF10A	Down	Up	Total
MCF10A_9 Gy CONV-RT vs Untreated	38	179	217
MCF10_15 Gy CONV-RT vs Untreated	58	335	393
MCF10_9 Gy FLASH-RT vs Untreated	51	233	355
MCF10_15 Gy FLASH-RT vs Untreated	67	448	515
MDA-MB-231	Down	Up	Total
MDA-MB-231_9 Gy CONV-RT vs Untreated	28	121	149
MDA-MB-231_15 Gy CONV-RT vs Untreated	29	198	227
MDA-MB-231_9 Gy FLASH-RT vs Untreated	29	240	269
MDA-MB-231_15 Gy FLASH-RT vs Untreated	25	263	288
FLASH-RT vs CONV-RT comparisons			
MCF10A	Down	Up	Total
MCF10_9 Gy FLASH-RT vs 9 Gy CONV-RT	0	18	18
MCF10_15 Gy FLASH-RT vs 15 Gy CONV-RT	0	6	6
MDA-MB-231	Down	Up	Total
MDA-MB-231_9 Gy FLASH-RT vs 9 Gy CONV-RT	0	13	13
MDA-MB-231_15 Gy FLASH-RT vs 15 Gy CONV-RT	0	0	0

**Table 6:** List of significant up-regulated genes in response to FLASH-RT, with respect to CONV-RT treatment in MCF10 and in MDA-MB-231 cells.

MCF10A 9 Gy FLASH vs CONV				
Symbol	Description	p-value	UP/DOWN regulation	Fold change of validated genes
*PRDM1*	PR/SET domain 1	5,6019E-17	UP	
*SOCS3*	suppressor of cytokine signaling 3	4,217E-16	UP	2,19
*TNFAIP3*	TNF alpha induced protein 3	2,078E-12	UP	2,06
*NR4A1*	nuclear receptor subfamily 4 group A member 1	2,1227E-08	UP	2,75
*IL6*	interleukin 6	3,7223E-06	UP	1,72
*NR4A2*	nuclear receptor subfamily 4 group A member 2	7,0621E-06	UP	1,86
*MT-CO2*	mitochondrially encoded cytochrome c oxidase II	1,5005E-05	UP	2,51
*MT-CYB*	mitochondrially encoded cytochrome b	1,9452E-05	UP	3,21
*MT-CO1*	mitochondrially encoded cytochrome c oxidase I	1,987E-05	UP	
*MT-ND4L*	mitochondrially encoded NADH:ubiquinone oxidoreductase core subunit 4L	6,0956E-05	UP	2,63
*MT-CO3*	mitochondrially encoded cytochrome c oxidase III	6,4526E-05	UP	2,48
*MT-ND2*	mitochondrially encoded NADH:ubiquinone oxidoreductase core subunit 2	8,3656E-05	UP	
*MT-ATP6*	mitochondrially encoded ATP synthase membrane subunit 6	9,6958E-05	UP	
*MT-ND1*	mitochondrially encoded NADH:ubiquinone oxidoreductase core subunit 1	0,00010321	UP	
*MT-ND4*	mitochondrially encoded NADH:ubiquinone oxidoreductase core subunit 4	0,00011121	UP	
*MT-ND5*	mitochondrially encoded NADH:ubiquinone oxidoreductase core subunit 5	0,00016675	UP	
*MYLIP*	myosin regulatory light chain interacting protein	0,00022364	UP	
*MT-ATP8*	mitochondrially encoded ATP synthase membrane subunit 8	0,00027481	UP	

MCF10A 15 Gy FLASH vs CONV				
Symbol	Description	p-value	UP/DOWN regulation	Fold change of validated genes
*MT-ND3*	mitochondrially encoded NADH:ubiquinone oxidoreductase core subunit 3	1,9842E-06	UP	2,42
*MT-CO1*	mitochondrially encoded cytochrome c oxidase I	1,0578E-05	UP	
*MT-CO3*	mitochondrially encoded cytochrome c oxidase III	5,0719E-05	UP	2,39
*MT-CO2*	mitochondrially encoded cytochrome c oxidase II	0,00014024	UP	3,55
*MT-CYB*	mitochondrially encoded cytochrome b	0,00024774	UP	2,26
*MT-ND4L*	mitochondrially encoded NADH:ubiquinone oxidoreductase core subunit 4L	0,00040769	UP	5,15

**Table 7:** List of significant up-regulated genes in response to FLASH-RT, with respect to CONV-RT treatment in MDA-MB-231 cells.

MDA-MB-231–9 Gy FLASH vs CONV				
Symbol	Description	p-value	UP/DOWN regulation	Fold change of validated genes
*MROH6*	maestro heat like repeat family member 6	1,3684E-07	UP	
*OBSCN*	obscurin, cytoskeletal calmodulin and titin-interacting RhoGEF	6,5172E-07	UP	1,83
*FBXW9*	F-box and WD repeat domain containing 9	3,8794E-06	UP	1,96
*TPM2*	tropomyosin 2	5,7254E-06	UP	
*PLXNB3*	plexin B3	7,8978E-06	UP	1,54
*MAPK8IP3*	mitogen-activated protein kinase 8 interacting protein 3	1,4091E-05	UP	
*ITGA10*	integrin subunit alpha 10	1,9627E-05	UP	
*TTLL3*	tubulin tyrosine ligase like 3	2,6779E-05	UP	2,56
*WDR27*	WD repeat domain 27	2,814E-05	UP	1,54
*IL18BP*	interleukin 18 binding protein	0,00021973	UP	
*CDK5RAP3*	CDK5 regulatory subunit associated protein 3	0,00032043	UP	1,88
*RNF207*	ring finger protein 207	0,00038352	UP	
*GOLGA8B*	golgin A8 family member B	0,00045795	UP	2,19

### 3.5 GSH/GSSG concentration

**Figure 7:**
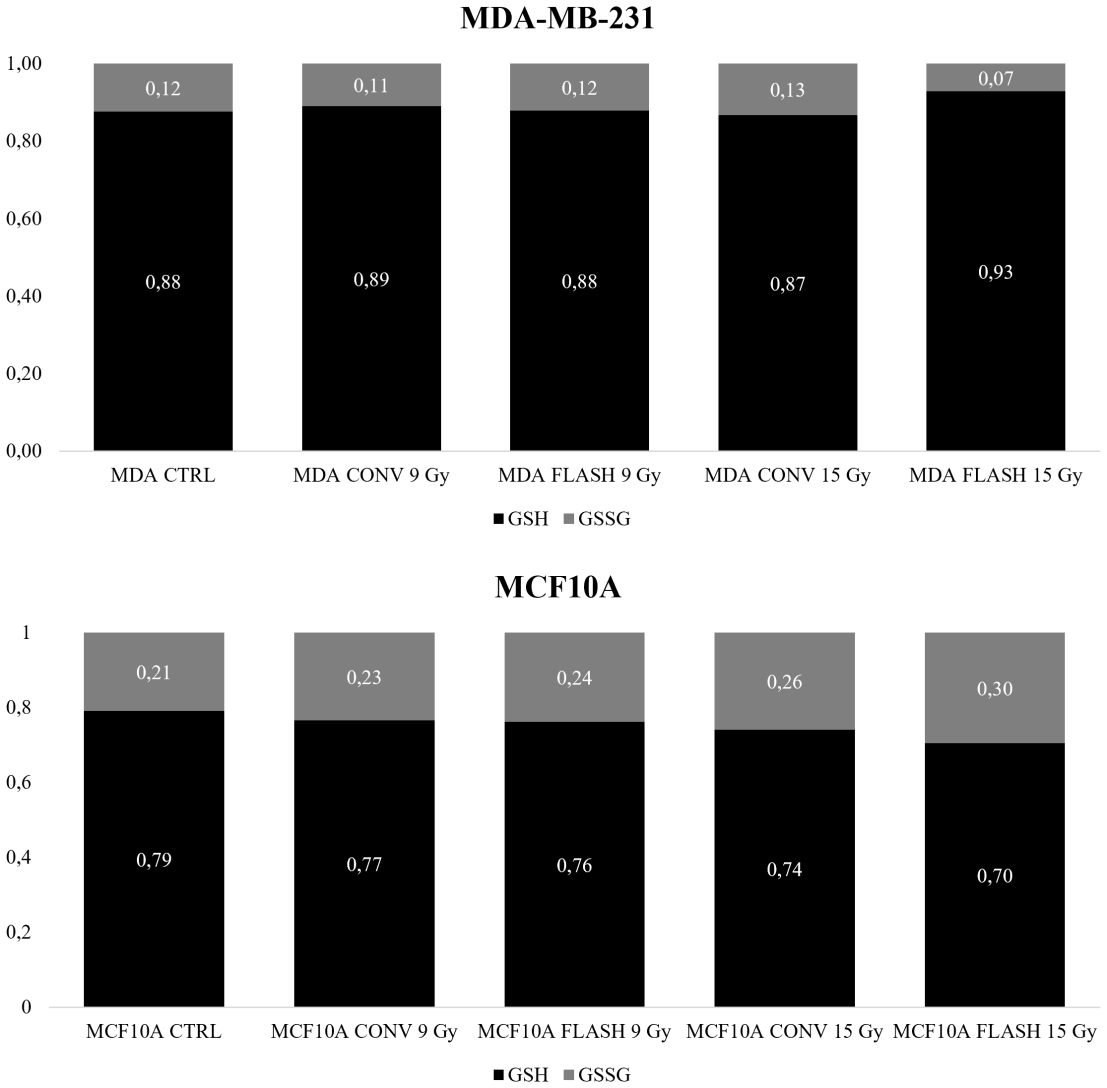
Percentage distribution of GSH/GSSG average intracellular content expressed in nmol/mg of total protein from 6 biological samples of MCF10A and MDA-MB-231 cell lines.

The GSH/GSSG ratio was quantified 24 hours post RT treatment, as above reported, in order to estimate differences among the cellular oxidation status due to the dose, or dose rate effects. **Figure 7** shows the percentage distribution of average intracellular GSH/GSSG content, expressed in nmol/mg of total protein from 6 biological replicates for each treatment modality.

At the time point chosen, no differences between untreated and treated samples were observed in both cell lines, neither with respect to the administered dose, 9 or 15 Gy, nor with respect to the different dose rate applied.

### 3.6 Immunological profiling

**Figure 8:**
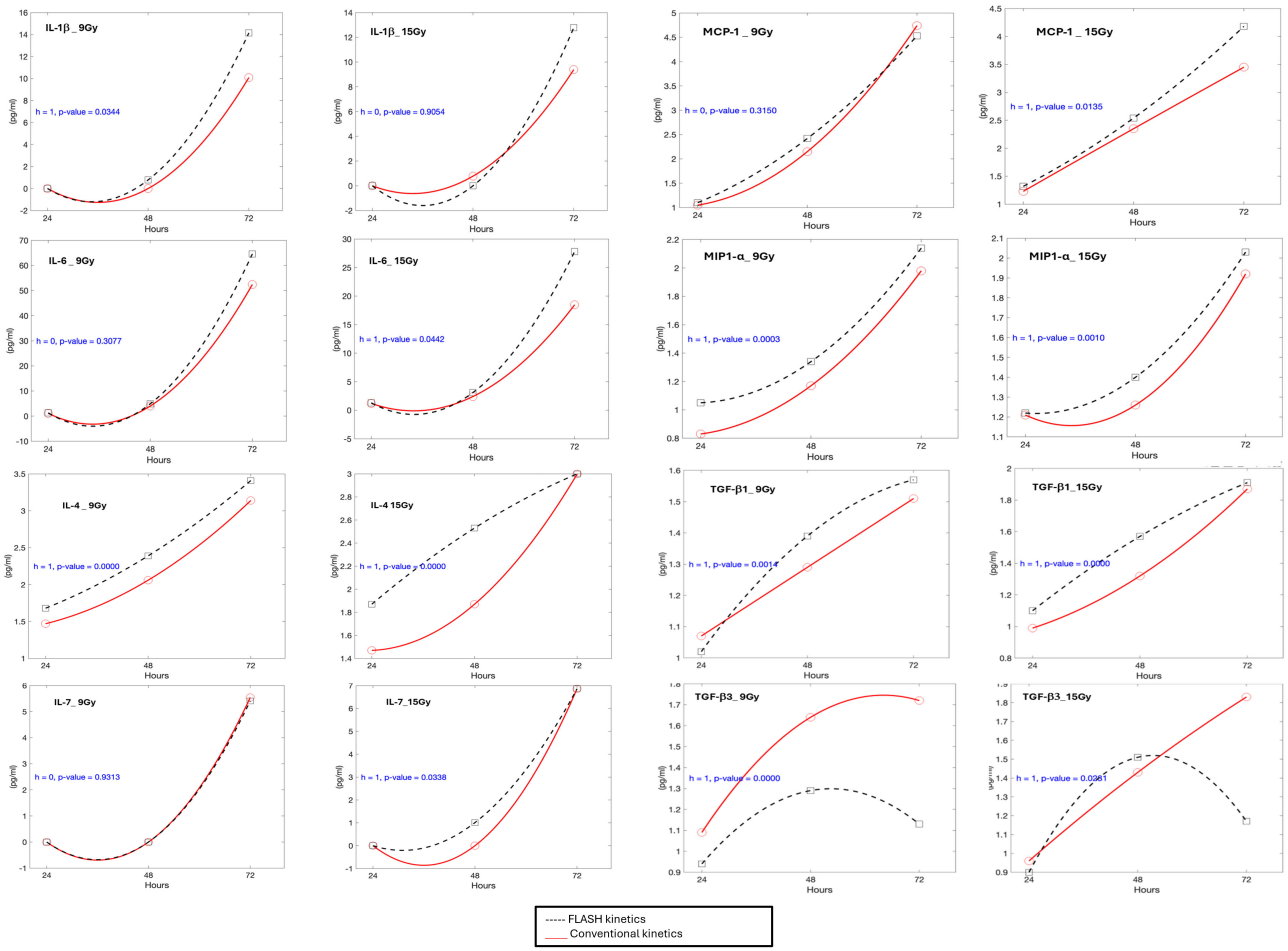
Representative panel of cytokine kinetic curves of expression in MDA-MB-231 subjected to CONV and FLASH irradiation with the doses of 9 and 15 Gy.

Differential immunological profiles were investigated by using Luminex approach on conditioned medium (CM) collected from untreated and treated MCF10A and MDA-MB-231 cells at the time points of 24, 48 and 72 hours post irradiation with the doses of 2, 9 and 15 Gy, delivered with CONV (6 Gy/min) or FLASH irradiation (230 Gy/s). **Tables 3** and **4** report normalized values of 20 cytokines, chemokines and growth factors, listed in the method sections, as means of duplicates for each protein and time point of treated sample, with respect to the means of duplicates for each protein/time point of untreated controls (time point 0). Some molecules were undetected in MCF10A cells (IL-1β, IL-4, IL-5, IL-7, IL-10, IL-12p70, IL-13, IL-17A, IFN-γ, MCP-1) and MDA-MB-231 cells (IL-5, IL-10, IL-12p70, IL-13, IL-17A, IFN-γ). The other detected molecules were upregulated in a time- and dose -dependent manner in both the cell lines, confirming the same kinetics of cytokine production already previously observed by our group (11, 14). Concerning the dose rate dependence, the MDA-MB-231 cell line showed a higher production of some inflammatory molecules (IL-1α, IL-1β, IL-6), chemokines (MCP-1, MIP-1α), immunomodulatory markers (IL-2, G-CSF) and TGF-β1, following irradiation with the FLASH dose rate at the time point of 72 hours, especially by using 15 Gy. The significant comparisons between non normalized mean values of cytokine concentration produced in response to CONV and FLASH-RT, are marked with an asterisk in **Tables 3** and **4**. The **Supplementary File 2** report table describing *p*-values and confidence intervals (C.I.) of significant comparisons. The 72-hour time point was featured by the detection of significantly higher concentrations of the above-reported markers after FLASH-RT modality. In addition, in order to describe the kinetics of cytokine production during the 24-, 48-and 72-hour time points, second-order polynomial curves were obtained by data fitting, and a *t-test* was applied to search for significant differences between CONV and FLASH kinetic curves. **Figure 8** displays a selection of the kinetic curves for those cytokines having significantly different expression profiles for at least one dose in MDA-MB-231 cells. The complete panel of expression kinetics of all cytokines tested in both cell lines MCF10A and MDA-MB-231 is reported in **Supplementary File 3**.

## 4 Discussion

Radiation-induced toxicity is due to cellular damage, inflammation and unbalanced redox status. In this context, the oxygen tissue tension has been proven to be tightly linked to tissue sparing effect, as the hypoxic condition results in being protective even using CONV dose rates (3, 4). In previous work, conducted on zebrafish embryos subjected to proton CONV (0.6 Gy/s) or FLASH (317 Gy/s) irradiation with a dose of 30 Gy, we reported a tissue-specific sparing effect, which was always significantly affected by hypoxic conditions, both applying CONV and FLASH dose rates. Conversely, not all the tissue targets had a significant gain when embryos were irradiated with FLASH respect to CONV dose rate, but it was observed only for the pericardial edema, irradiated under normoxic conditions, and for head and eye size under hypoxic conditions (4). Thus, the dose rate effect cannot be univocally explained for all the tissue targets, nor is oxygen the unique key regulating factor.

In this perspective, although the *in vitro* studies collected till now have generally produced few insights on FLASH effect biological mechanisms, we investigated the effects of FLASH (230 Gy/sec) in comparison with CONV electron irradiation (6 Gy/min) under normoxic irradiation, on two cell breast lines, the MCF10A and the MDA-MB-231, that previously we have deeply investigated for their survival, genomic and immune response to CONV irradiation by using electrons or proton beams using 9 Gy and 15 Gy. Our results support evidence that the response to radiation treatments is driven by gene expression in a cell line-dependent manner (10–16).

In the present study, we first analyzed the survival curves of cells subjected to CONV and FLASH-RT in normoxic conditions, but we did not observe significant differences in cell survival between the two irradiation modalities in both cell lines, in line with several literature findings (33). As regards cell survival experiments, the group of Adrian et al. studied the survival curves of different cell lines, irradiated under normoxic or hypoxic conditions. They showed oxygen-dependent sparing effect on the DU-145 cell line treated at O_2_ levels < of 4,4% by using a dose-rate of 600 Gy/s (34). Instead, in normoxia, a higher dose rate of 800 Gy/s was necessary to observe the FLASH effect (6).

Secondly, we assessed the DNA damage kinetic repair at 30 minutes, 1 hour, and 24 hours post-irradiation at 2 and 5 Gy by quantifying the number of γ-H2AX and 53BP1 foci. In this case, we observed a dose effect on the MDA-MB-231 cell line, where a greater persistence of 53BP1 foci, referable to double-strand break damage, was still observable 24h post-treatment with the dose of 5 Gy. Instead, a dose rate effect was referable to a significantly greater quantity of γ-H2AX foci 1-hour post-treatment with 5 Gy in both the cell lines subjected to FLASH-RT, compared to CONV irradiation, a difference not more observable at 24 hours post-treatment. Similarly to our finding, Adrian G. and colleagues obtained no differences in the 53BP1 foci count on MDA-MB-231 and the other two cell lines after 3 Gy of electron irradiation with the dose rate of 800 Gy/s, at 2 and 24 hours post treatment (6). Fouilade C. et al. found no difference in γ-H2AX foci, but significantly less 53BP1 ones in FLASH vs CONV treated fibroblast cell lines (MRC5 and IMR-90), but not in the A589 tumor cells, using approximately 5 Gy in normoxia (7). Although the literature is conflicting, our results indicate an increase in DNA damage induced by FLASH irradiation at a given timepoint, as evidenced by the early appearance of a greater number of γ-H2AX foci; however, this increase is likely not sufficient to generate subsequent functional effects, like cell killing.

Regarding the genomic profiles of the two treated cell lines, we can resume our finding in three important observations: 1) FLASH irradiation induced a greater number of deregulated genes in both the cell lines, especially with the higher dose of 15 Gy (**Table 5**); 2) with some exceptions, the common 9∩15 Gy list of deregulated pathways was different between CONV and FLASH irradiation for both the cell lines (**Figure 6**); 3) following the direct bioinformatic comparison of FLASH vs CONV gene lists for each dose, only qualitative differences were highlighted (**Tables 6**, **7**). Specifically, FLASH-RT was able to upregulate the mitochondrial gene expression in the non-tumorigenic MCF10A cells, whereas it induced upregulation of structural genes in the tumorigenic MDA-MB-231 cells.

Thus, our study shows that the dose rate effect is perceived as a more intense stimulus by both cell lines, able to enhance gene expression. In this regard, it is interesting that an in-silico study shows that FLASH-RT is able to generate higher pressures, opening to the fact that some biological observations related to FLASH effects could be due to the mechanical impact of acoustic waves into the cells (35). In this perspective, it could be probably justified also our observation regarding the 9 Gy FLASH induced MDA-MB-231 gene expression signature with respect to CONV irradiation, which shows the up regulation of structural and signal transduction genes (**Tables 6**, **7**). Given these results, we investigated the microtubule fibers integrity in MDA-MB-231 cells, as biological target of IR with self-renewal ability (36).

The imaging results by confocal microscopy showed a significant minor microtubule damage under 9 Gy FLASH-RT with respect to CONV in MDA-MB-231 cells. However, the mechanisms involved need to be further explored. Furthermore, our findings, which demonstrate an up-regulation of mitochondrial genes in non-tumorigenic MCF10A cells subjected to FLASH-RT, confirm the central role of mitochondria preservation in the FLASH effect explanation. In this regard, Dahl et al. (37) reported increased MT-DNA copy number in B6N mice subjected to FLASH-RT. In our FLASH irradiated MCF10A cells, we observed the specific upregulation of genes involved in the respiratory chain maintenance and in the oxidative stress control, thus protecting mitochondria for the maintenance of aerobic metabolism and oxidative stress control and lowering the risk of intrinsic apoptosis.

Thus, to better understand the oxidative stress status of the two cell lines, we tested the GSH/GSSG ratio 24 hours post- irradiations with the two different dose-rates. Interestingly, we did not observe significant differences in the glutathione balance in FLASH vs CONV-RT in both cell lines. This observation is in line with the idea that FLASH irradiation does not increase ROS levels but can rather differently modulate the rate of ROS species production in the physico-chemical stages and their balances with respect to CONV one (3). Indeed, experimental measurements showed that the dose rate increase produces H_2_O_2_ and ΔpO_2_ decrease and e_aq-_ increase (1, 38, 39).

The differences found in our early observation of major indirect DNA damage in FLASH vs CONV- irradiated cells, detected 1 hour post treatment with increased γ-H2AX foci (which disappeared at 24 hours), could be related to a shift in the accumulation of the damage in the FLASH conditions as compared to the conventional ones. Indeed, this would lead to a mismatch of the two peaks of the damage in the temporal coordinate, with some timepoints showing a larger response in FLASH and some others the opposite, until the levelling out of the effect in the residual damage. This effect would be consistent with the mechanistic hypothesis of the peroxyd radical recombination, introduced by Labarbe et al. (40), confirmed in our previous work (41), and in general with the various literature data reporting a difference in damage at a given timepoint, which disappears at later times (40, 42). Several data, indeed, report a diminution in DNA damage after UHDR radiation, mostly in simplified systems as plasmids (43, 44), where repair doesn’t make an interplay, and thus the connection with the ROO. Saturation may be seen more directly, while data in cells have a more complex pathway (40, 42). A verification for this hypothesis may be done, however, only with a denser and finer resolution of temporal measurements, which are out of the scope of the present paper but are envisaged for future work. It is also possible that GSSH/GSSG ratio alteration could be observable at earlier time points; however, we tested cells at 24 hours, according to the gene expression time point evaluation.

Finally, we analysed the immunological profiling in response to the two irradiation modalities. Previously, we characterized these two cell lines for their immune secretome in response to electron and proton irradiation in respect to untread cells, and we showed no difference regarding the irradiation type used, but rather a cell- and dose- dependent profiling, with typical kinetics of molecules productions up to 72 hours post- treatment (11, 14). In particular, we previously observed the tumorigenic MDA-MB-231 cells’ capacity to produce a broader panel of molecules consistent with a stronger pro-inflammatory profile and a TH1-type response activation, compared to MCF10A cells. In view of these previous findings, we compared CONV and FLASH irradiation and observed no qualitative differences in the panel of molecules produced in response to the two different dose rates in both cell lines. Moreover, MDA-MB-231 cells displayed a specific ability to produce higher levels of several immunological markers, among those assessed, in response to FLASH-RT compared with CONV. Specifically, we detected a higher production of some inflammatory factors (IL-1α, IL-1β, IL-6), chemokines (MCP-1, MIP-1α), immunomodulatory markers (IL-2, G-CSF) and TGF-β1, following FLASH irradiation with peaks of production at the time point of 72 hours at the dose of 15 Gy, some of which resulted significantly different between the two dose-rates (**Table 3**). On the contrary, the MCF10A cells did not show major differences in the production of immunological molecules when subjected to the two different dose-rates. In addition, as shown in **Figure 8**, some cytokines showed significantly different kinetics of production in MDA-MB-231 cells. In particular, the pro-inflammatory IL-1β, IL-6, MCP-1, MIP-1α, and IL-7 and the anti-inflammatory markers IL-4 and TGF-β1 were significantly upregulated, whereas TGF-β3 was significantly downregulated by FLASH-RT. These results are in line with the observation of an overall higher level of deregulated genes in FLASH-irradiated cells, suggesting that they receive a major stimulus to increase gene and protein expression.

## 5 Conclusion

The FLASH effect mechanism is still a biological puzzle where some pieces are going to be inserted at the right site. Our study provided novel insights into the early biological effects induced by very high dose-rates in normoxic conditions, within 24 hours post-treatment. Specifically, the DNA repair kinetics showed significantly greater γ-H2AX foci within 1 hour post treatment with 5 Gy of FLASH-RT in the MDA-MB-231 and MCF10A cell lines, which was reversed later at 24 hours. This observation, together with the specific healthy MCF10A transcriptomic response to FLASH-RT, featured by the upregulation of mitochondrial genes, revealed an early response to oxidative stress caused by FLASH-RT. However, the GSH/GSSG balance was not affected at 24 hours in both cell lines. In addition, we observed an increased rate of gene expression and cytokine production in response to FLASH-RT. However, no difference in survival curves was obtained after 10 days post treatment by clonogenic assay, suggesting that the dose-rate effect may modulate the early cellular response.

## Data Availability

The authors acknowledge that the data presented in this study must be deposited and made publicly available in an acceptable repository, prior to publication. Frontiers cannot accept an article that does not adhere to our open data policies.
