# Supplementary material for: Early molecular changes induced by FLASH irradiation in MCF10A and MDA-MB-231 breast cell lines
Source: Front Oncol. 2026 Jul 28;16:1857611. doi: 10.3389/fonc.2026.1857611 (PMC13457129; doi:10.3389/fonc.2026.1857611)
Supplement: Supplementary file 1 [file SupplementaryFile1.pdf]

Supplementary file 1. List of used primers used for q-PCR.

Gene symbol	Gene name	Forward Primer 5′>3′	Reverse Primer 5′>3′
SOCS3	suppressor of cytokine signaling 3	AGGACGGAGACTTCGATTCG	AGCTGAAGGTCTTGAGGCG
TNFAIP3	TNF alpha induced protein 3	CTTCCACAGACACACCCATG	AAAGGGGCGAAATTGGAACC
IL6	interleukin 6	GAGACTTGCCTGGTGAAAATCA	GTTGGGTCAGGGGTGGTTAT
NR4A1	nuclear receptor subfamily 4 group A member 1	ACCTTCAAAACCCAAGCAGCC	ATCCCCAGCATCTTCCTTCC
NR4A2	nuclear receptor subfamily 4 group A member 2	AAGGAGGAGATTGGACAGGC	CATACTGCGCCTGAACACAAG
MT-ND3	mitochondrially encoded NADH:ubiquinone oxidoreductase core subunit 3	ACCACAACTCAACGGCTACA	TAGGGCTCATGGTAGGGGTA
MT-CO3	mitochondrially encoded cytochrome c oxidase III	CCCGCTAAATCCCCTAGAAG	GGAAGCCTGTGGCTACAAAA
MT-CO2	mitochondrially encoded cytochrome c oxidase II	CCATCCCTACGCATCCTTTA	GGTCGCCTGGTTCTAGGAAT
MT-CYB	mitochondrially encoded cytochrome b	GCCCTCGGCTTACTTCTCTT	GACGGATCGGAGAATTGTGT
MT-ND4L	mitochondrially encoded NADH:ubiquinone oxidoreductase core subunit 4L	TCGCTCACACCTCATATCCTC	GGCCATATGTGTTGGAGATTG
OBSCN	obscurin, cytoskeletal calmodulin and titin-interacting RhoGEF	AAACCTCCTCCGAAAGCTCC	GAGATGGTTAGTGTGTGGCG
FBXW9	F-box and WD repeat domain containing 9	GAAGAACTTTGACTGGCCGG	GGTCCCACAAGTTGACGTTG
PLXNB3	plexin B3	CATCGCTTCTCCGCACCTAAT	GGTTGGCATTGTCAGTGAGCT
TTLL3	tubulin tyrosine ligase like 3	TACAATCCAAGGCTGCTACCC	GGAACTCCTCGTCCTCATCT
WDR27	WD repeat domain 27	CTGTCATGGGCTCGCTTTTG	GGGACTTTGTGGCGGTTATTA
CDK5RAP3	CDK5 regulatory subunit associated protein 3	GCACTGCAGCCTGAAATGG	ATTCTTCGTGGAGGCCTCTG
GOLGA8B	golgin A8 family member B	GCACCGTCCCCAAAAGATTT	GGGCTGGTGAAGGAGTTTTC
WDR27	WD repeat domain 27	CTGTCATGGGCTCGCTTTTG	GGGACTTTGTGGCGGTTATTA
