# Supplementary material for: Early molecular changes induced by FLASH irradiation in MCF10A and MDA-MB-231 breast cell lines
Source: Front Oncol. 2026 Jul 28;16:1857611. doi: 10.3389/fonc.2026.1857611 (PMC13457129; doi:10.3389/fonc.2026.1857611)
Supplement: Supplementary file 2 [file SupplementaryFile3.pdf]

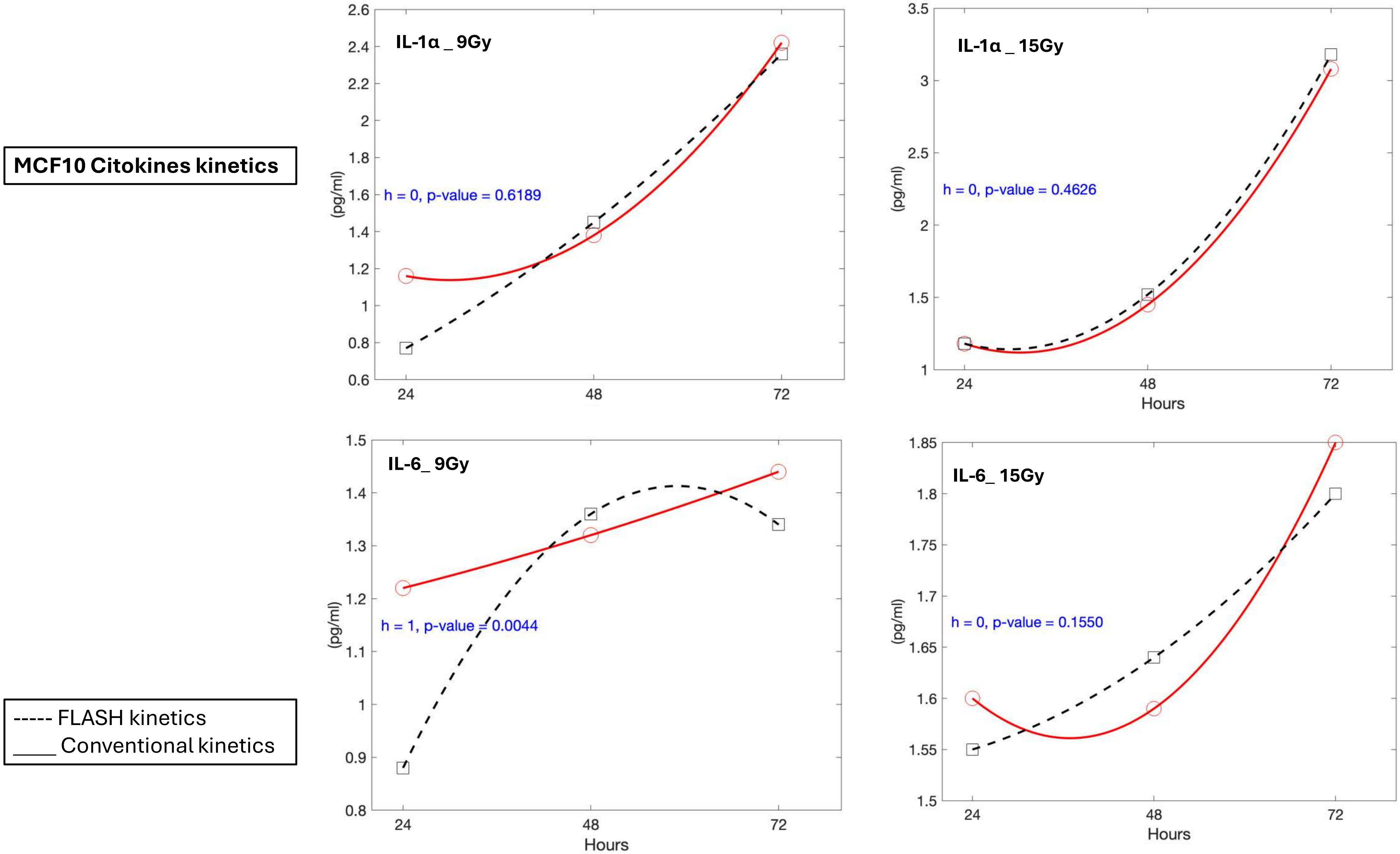

MCF10 Citokines kinetics
----- FLASH kinetics
____ Conventional kinetics
IL-1α _ 9Gy
h = 0, p-value = 0.6189
IL-1α _ 15Gy
h = 0, p-value = 0.4626
IL-6_ 9Gy
h = 1, p-value = 0.0044
IL-6_ 15Gy
h = 0, p-value = 0.1550
(pg/ml)
Hours
24
48
72

MCF10 Citokines kinetics

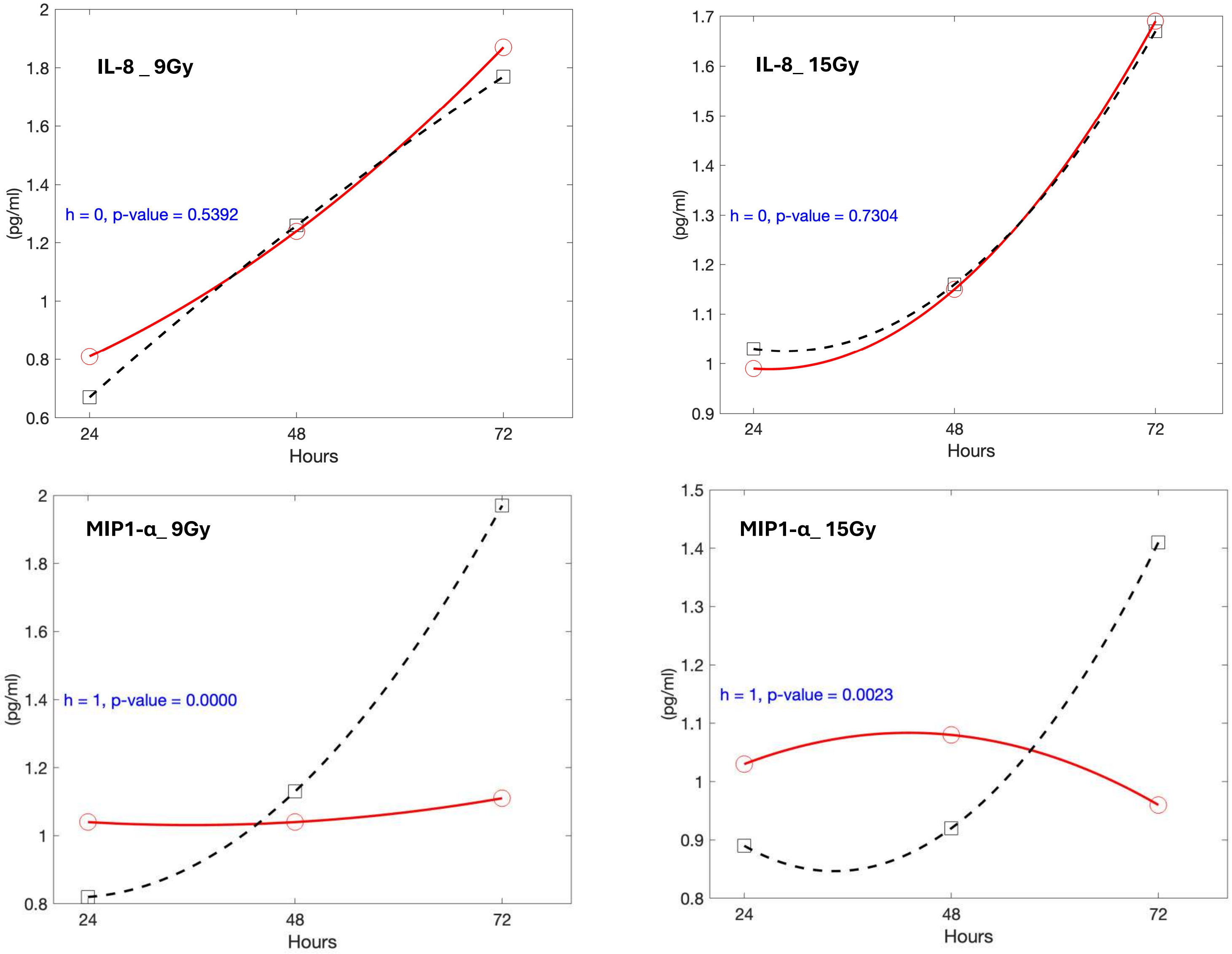

----- FLASH kinetics
____ Conventional kinetics

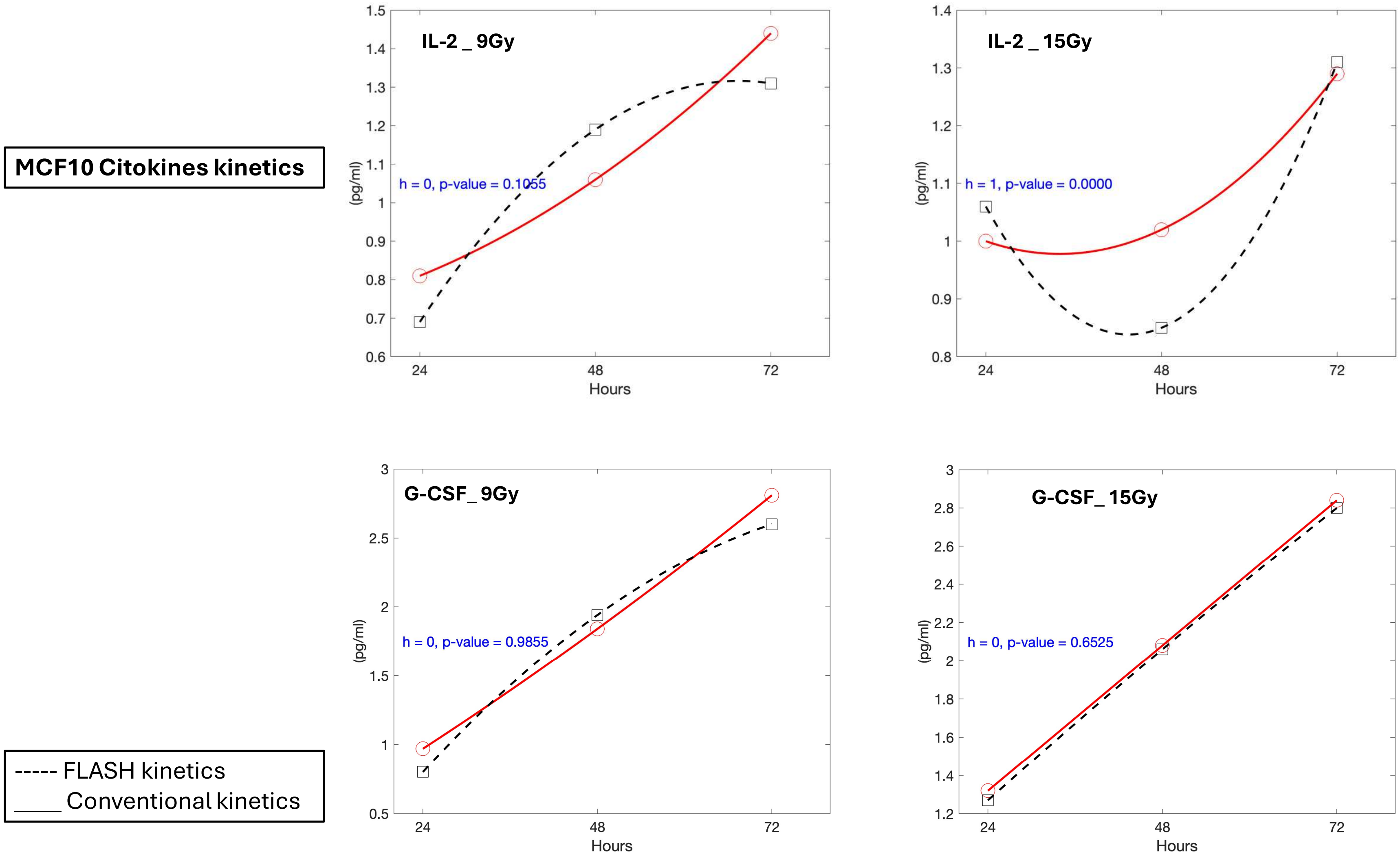
MCF10 Citokines kinetics
IL-2 _ 9Gy
h = 0, p-value = 0.1055
IL-2 _ 15Gy
h = 1, p-value = 0.0000
G-CSF_9Gy
h = 0, p-value = 0.9855
G-CSF_15Gy
h = 0, p-value = 0.6525
(pg/ml)
Hours
24
48
72
----- FLASH kinetics
____ Conventional kinetics

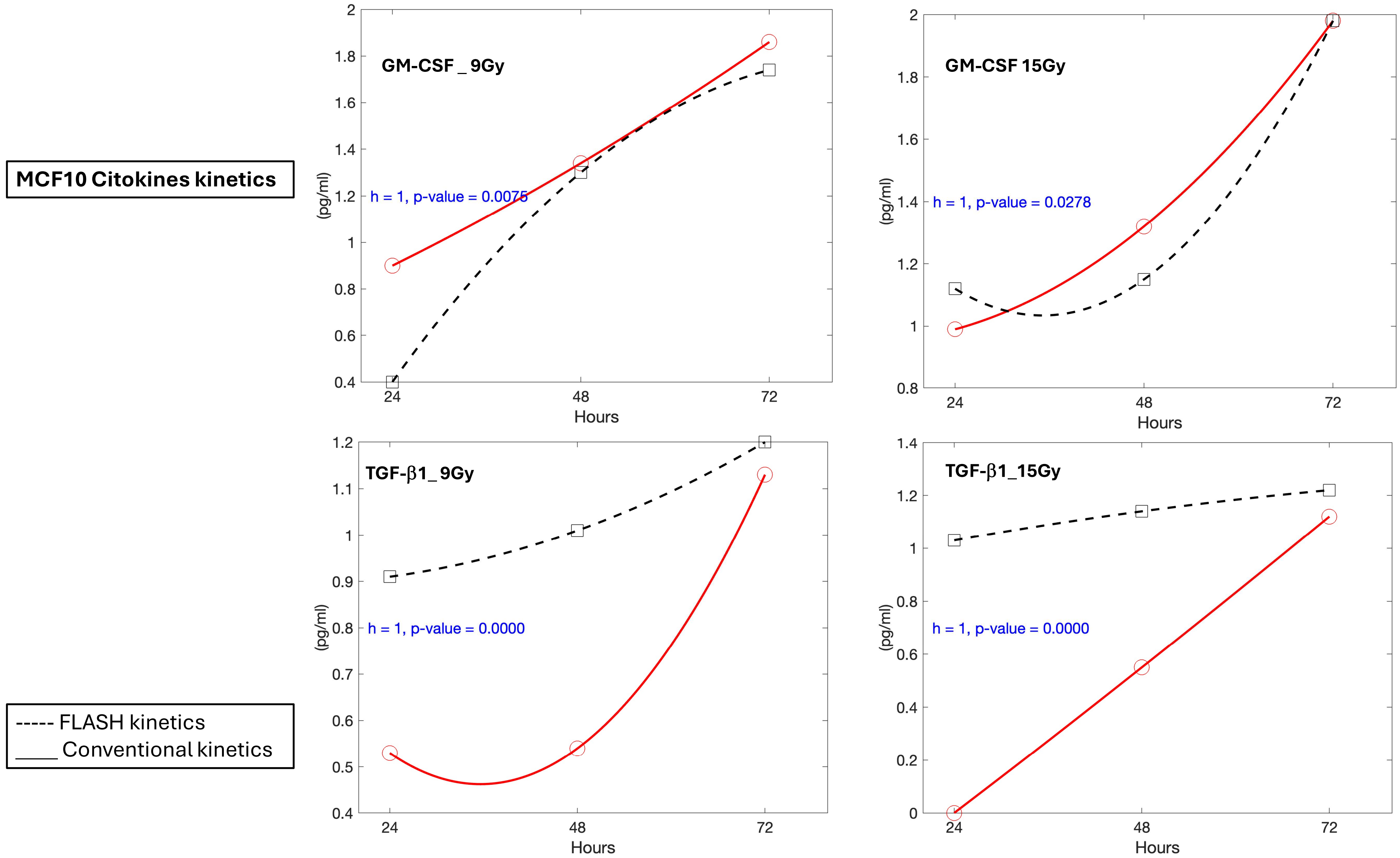
MCF10 Citokines kinetics
----- FLASH kinetics
____ Conventional kinetics
GM-CSF _ 9Gy
h = 1, p-value = 0.0075
(pg/ml)
Hours
GM-CSF 15Gy
h = 1, p-value = 0.0278
(pg/ml)
Hours
TGF-β1_ 9Gy
h = 1, p-value = 0.0000
(pg/ml)
Hours
TGF-β1_15Gy
h = 1, p-value = 0.0000
(pg/ml)
Hours

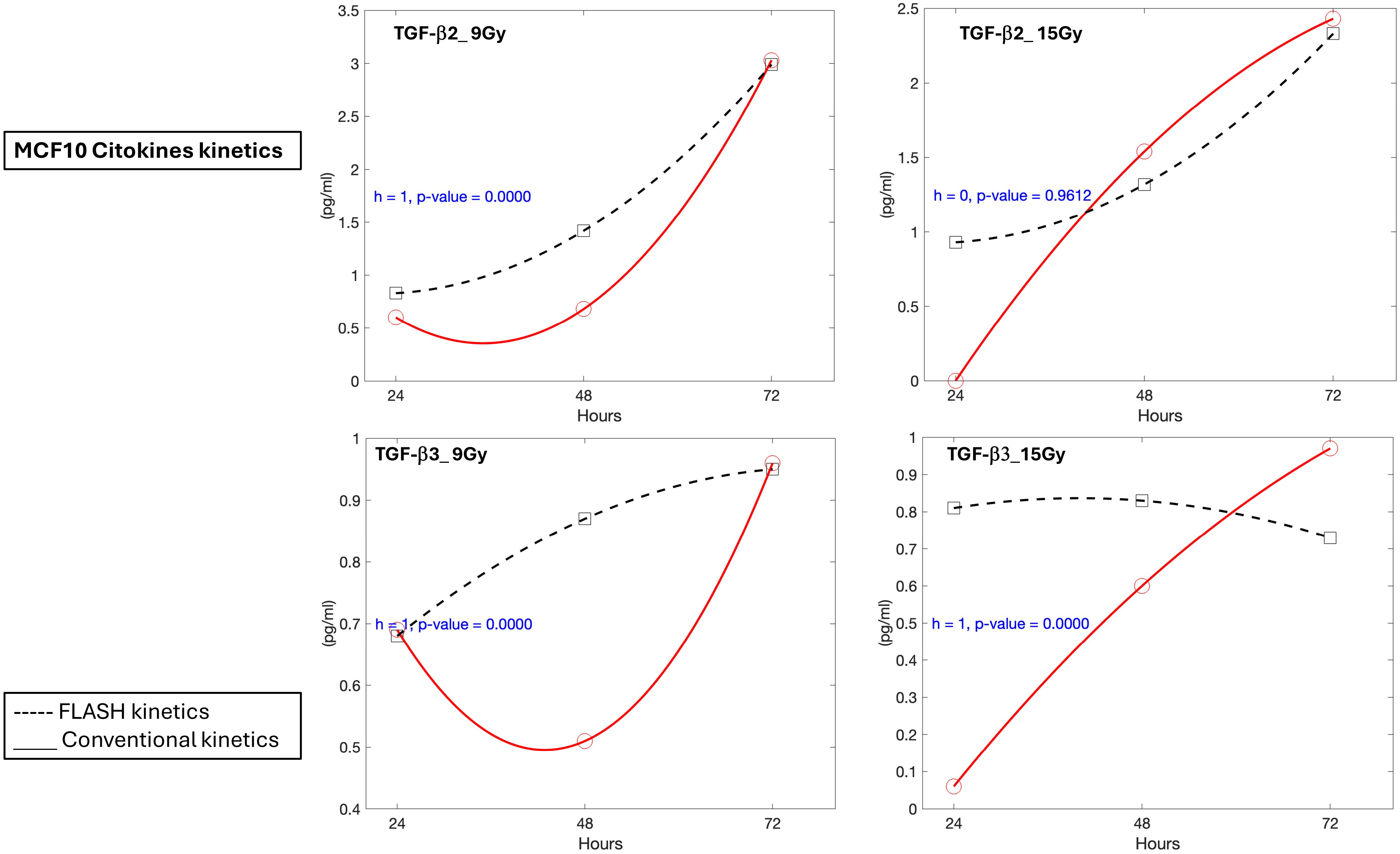
MCF10 Citokines kinetics
----- FLASH kinetics
____ Conventional kinetics
TGF-β2_ 9Gy
h = 1, p-value = 0.0000
TGF-β2_ 15Gy
h = 0, p-value = 0.9612
TGF-β3_ 9Gy
h = 1, p-value = 0.0000
TGF-β3_15Gy
h = 1, p-value = 0.0000
(pg/ml)
Hours
24
48
72

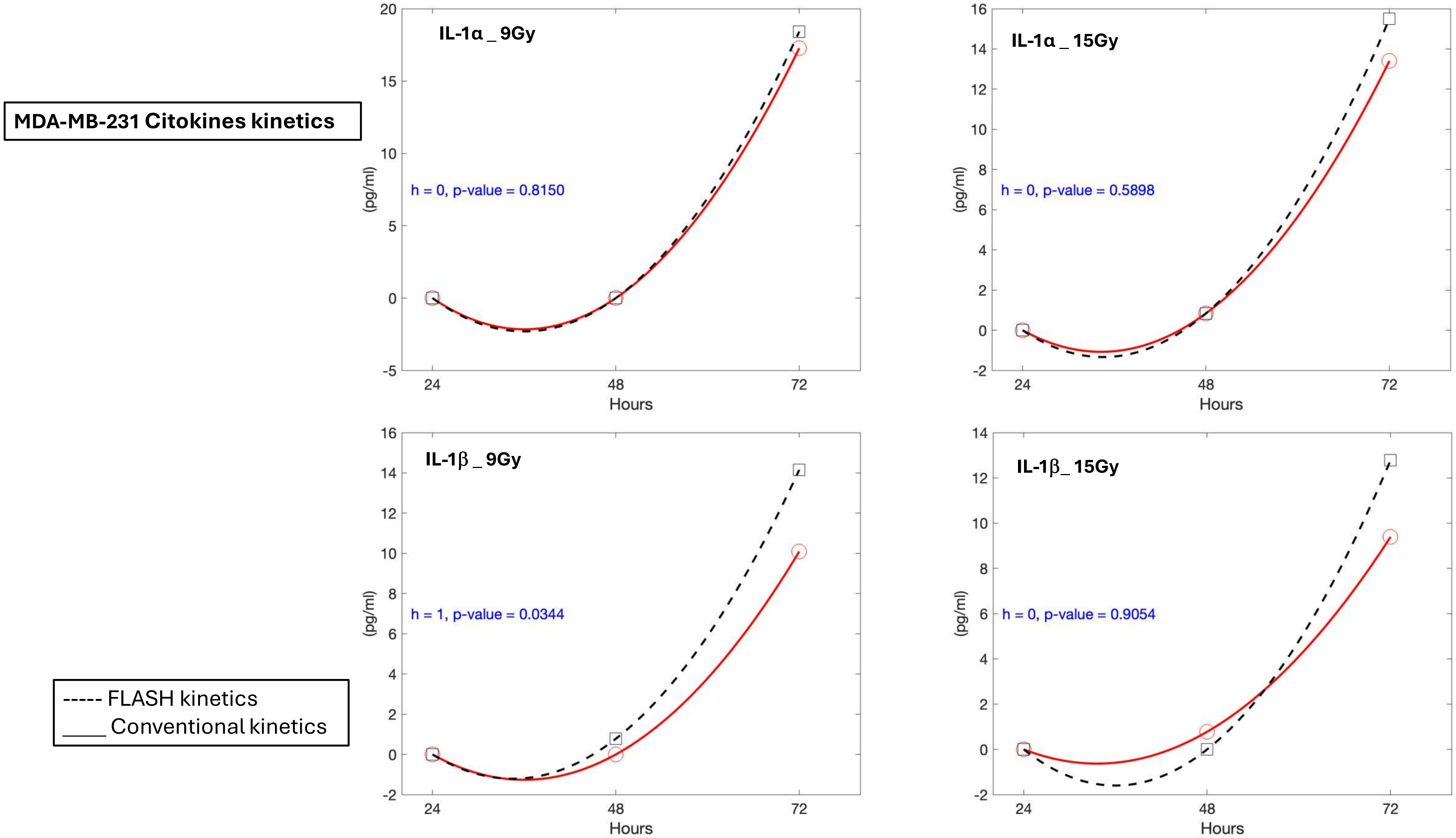

MDA-MB-231 Citokines kinetics
IL-1α _ 9Gy
h = 0, p-value = 0.8150
IL-1α _ 15Gy
h = 0, p-value = 0.5898
IL-1β _ 9Gy
h = 1, p-value = 0.0344
IL-1β_ 15Gy
h = 0, p-value = 0.9054
(pg/ml)
Hours
24
48
72
----- FLASH kinetics
____ Conventional kinetics

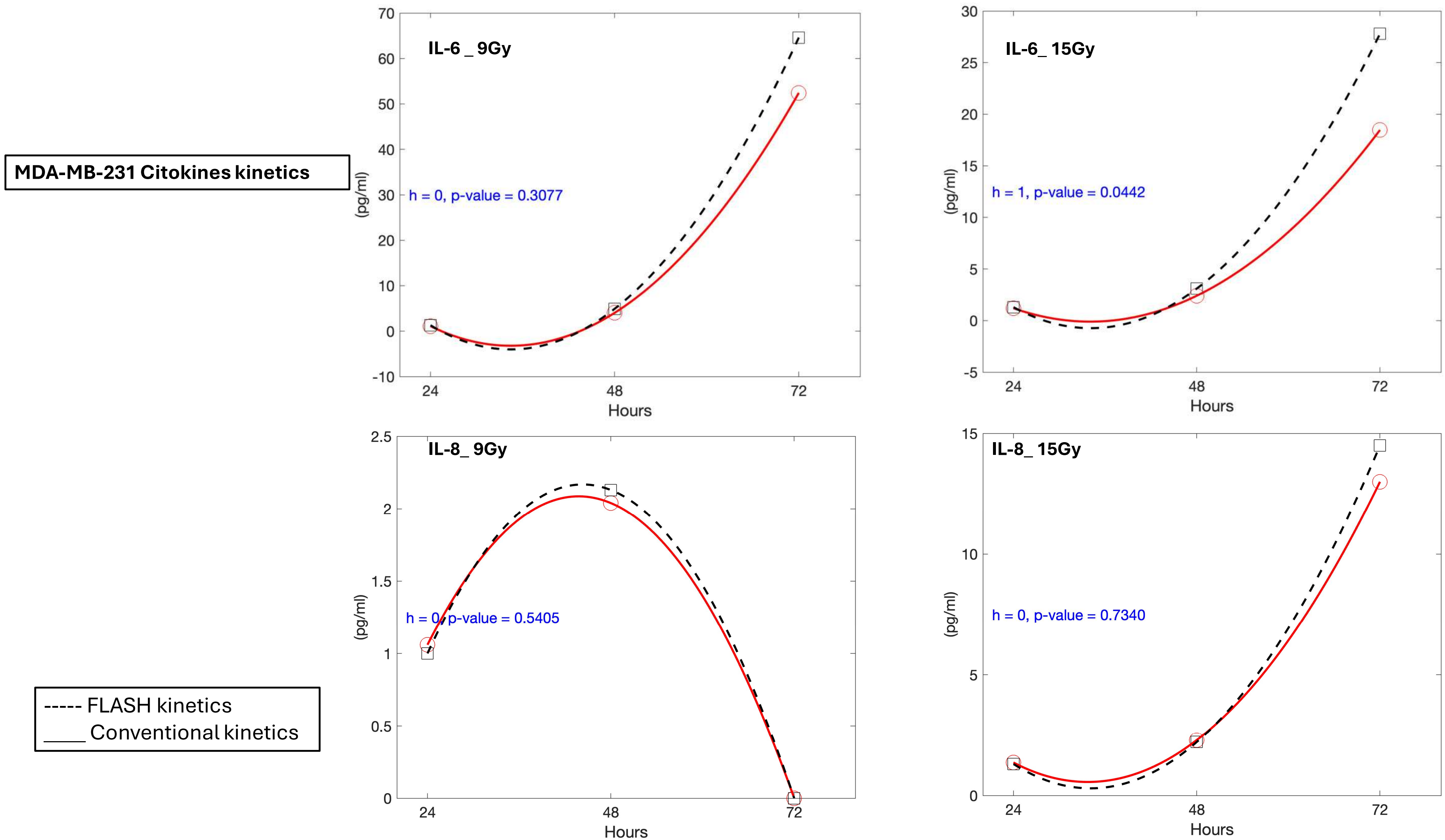
MDA-MB-231 Citokines kinetics
IL-6 _ 9Gy
h = 0, p-value = 0.3077
IL-6_ 15Gy
h = 1, p-value = 0.0442
IL-8_ 9Gy
h = 0, p-value = 0.5405
IL-8_ 15Gy
h = 0, p-value = 0.7340
(pg/ml)
Hours
24
48
72
----- FLASH kinetics
____ Conventional kinetics

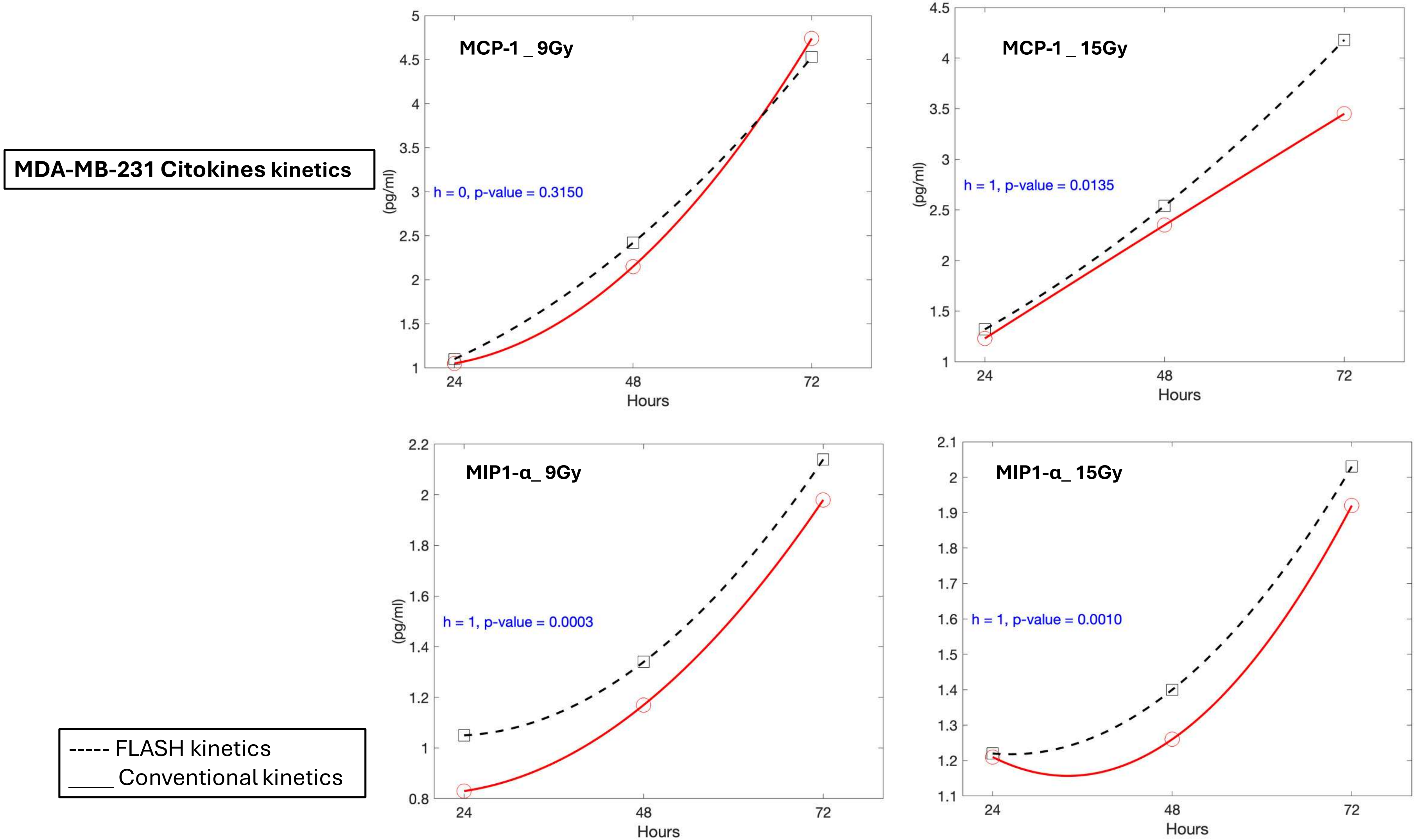
MDA-MB-231 Citokines kinetics
MCP-1 _ 9Gy
h = 0, p-value = 0.3150
MCP-1 _ 15Gy
h = 1, p-value = 0.0135
MIP1-α_ 9Gy
h = 1, p-value = 0.0003
MIP1-α_ 15Gy
h = 1, p-value = 0.0010
(pg/ml)
Hours
24
48
72
----- FLASH kinetics
____ Conventional kinetics

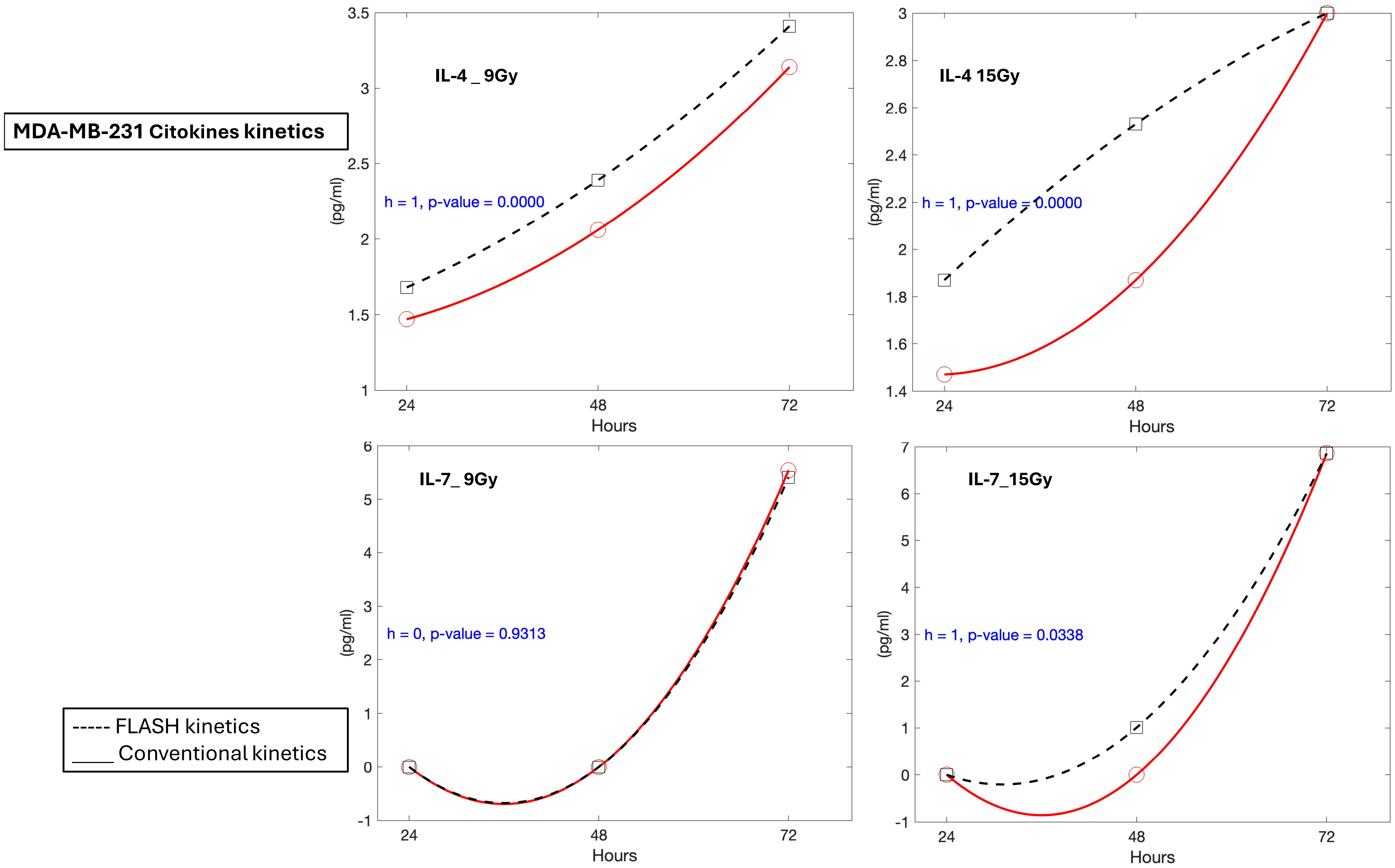
MDA-MB-231 Citokines kinetics
IL-4 _ 9Gy
h = 1, p-value = 0.0000
IL-4 15Gy
h = 1, p-value = 0.0000
IL-7_ 9Gy
h = 0, p-value = 0.9313
IL-7_15Gy
h = 1, p-value = 0.0338
(pg/ml)
Hours
24
48
72
----- FLASH kinetics
____ Conventional kinetics

MDA-MB-231 Citokines kinetics

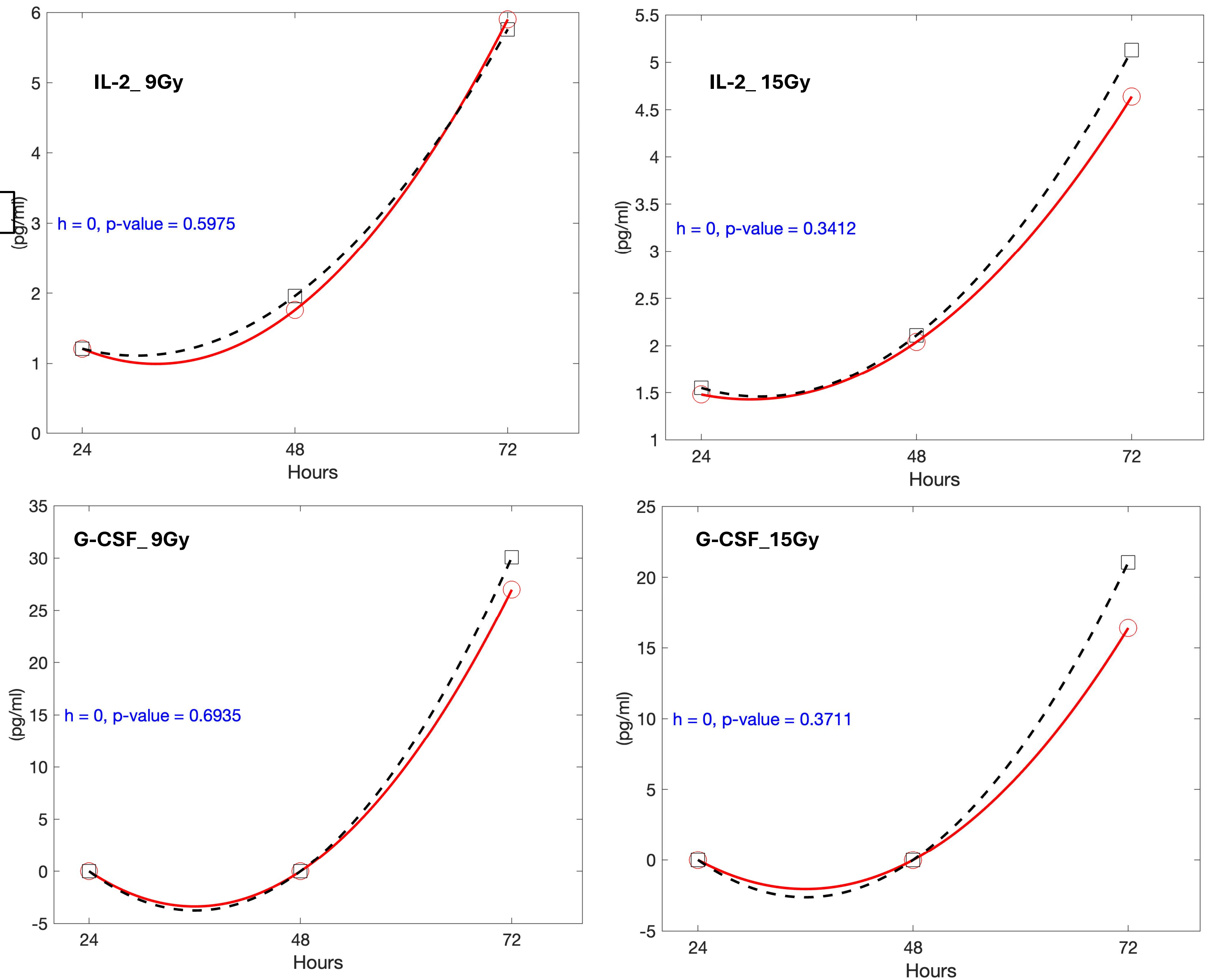

----- FLASH kinetics
____ Conventional kinetics

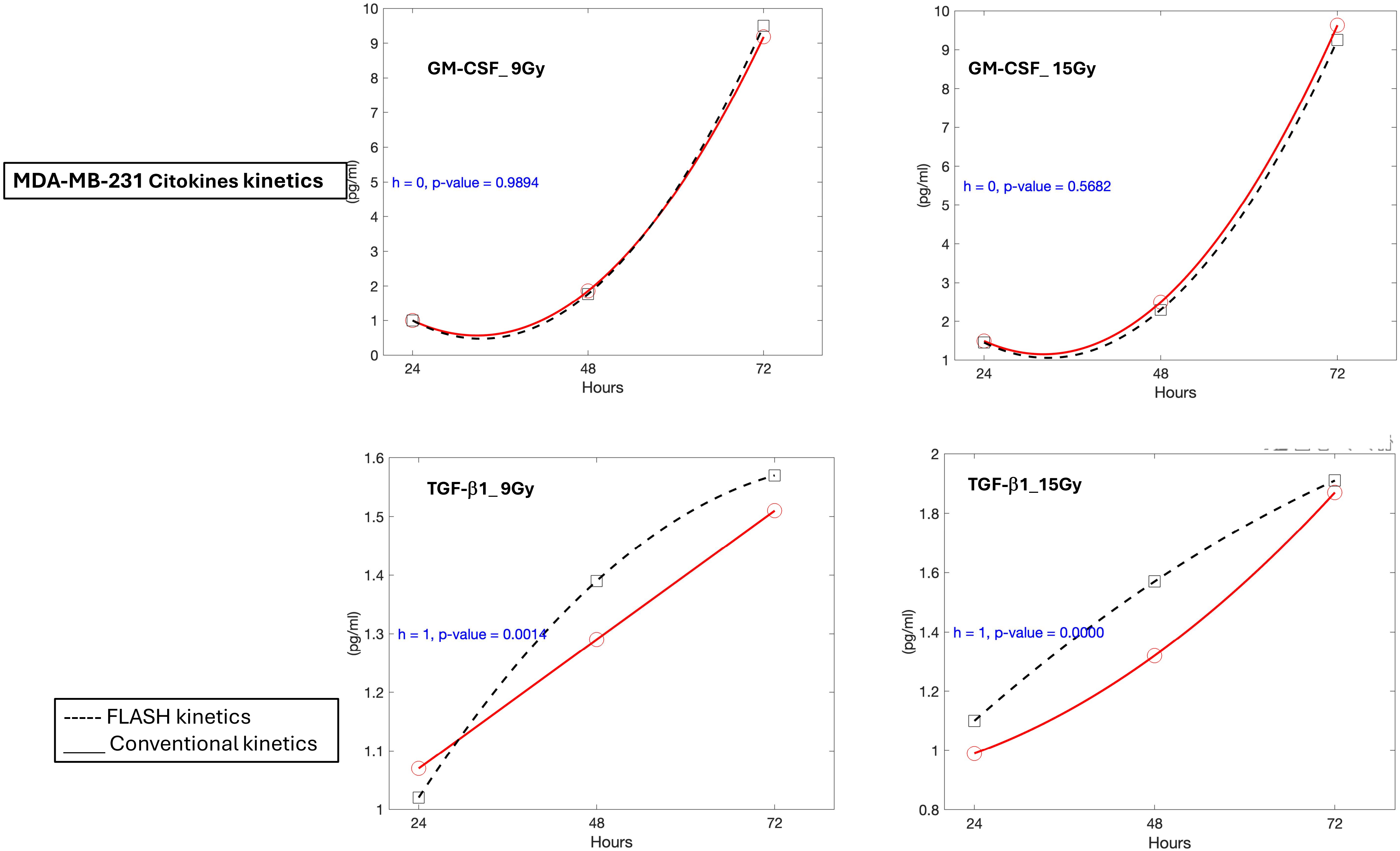
MDA-MB-231 Citokines kinetics
GM-CSF_ 9Gy
h = 0, p-value = 0.9894
GM-CSF_ 15Gy
h = 0, p-value = 0.5682
TGF-β1_9Gy
h = 1, p-value = 0.0014
TGF-β1_15Gy
h = 1, p-value = 0.0000
(pg/ml)
Hours
24
48
72
----- FLASH kinetics
____ Conventional kinetics

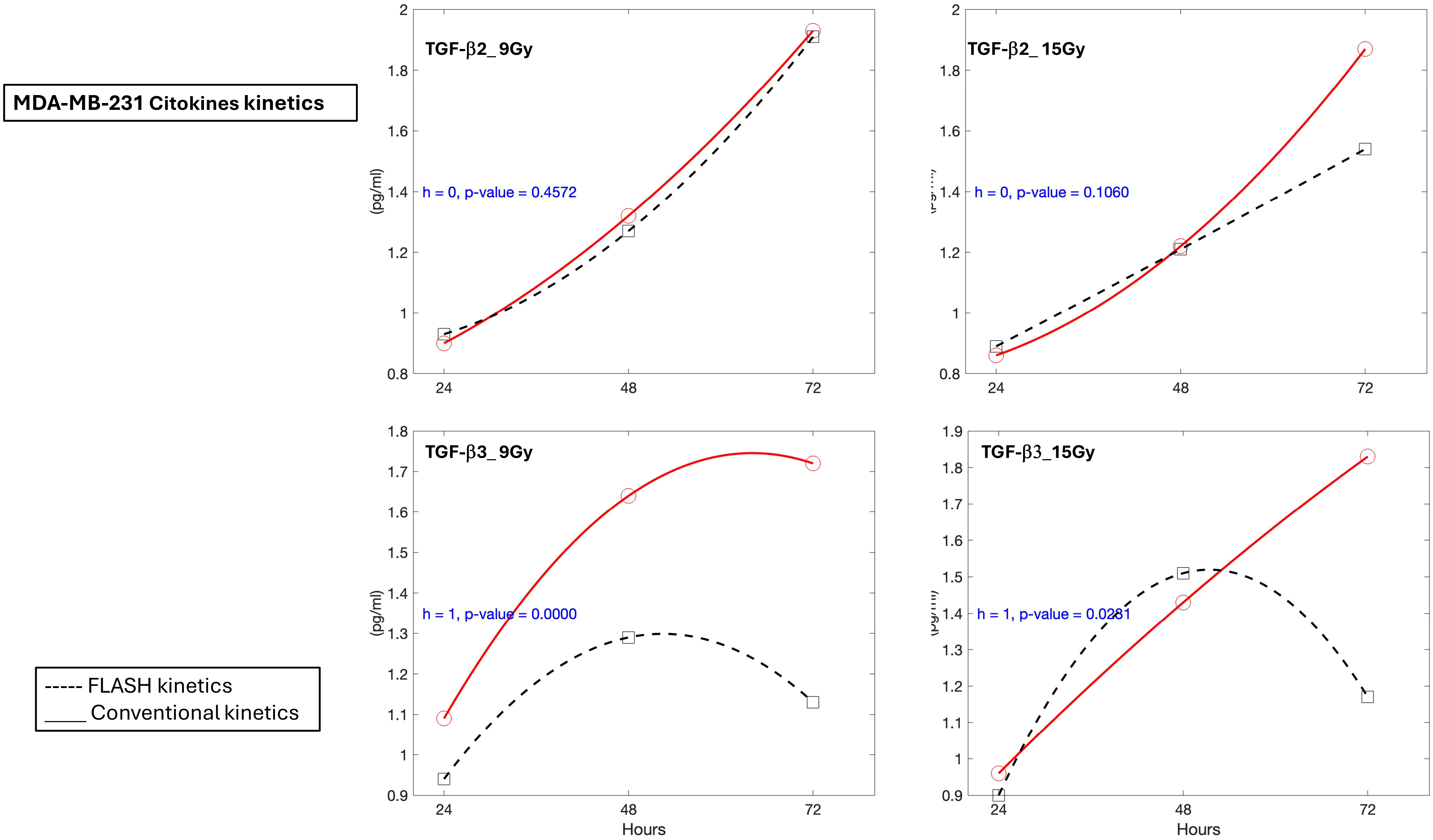
MDA-MB-231 Citokines kinetics
TGF-β2_ 9Gy
h = 0, p-value = 0.4572
TGF-β2_ 15Gy
h = 0, p-value = 0.1060
TGF-β3_ 9Gy
h = 1, p-value = 0.0000
TGF-β3_15Gy
h = 1, p-value = 0.0281
(pg/ml)
Hours
24
48
72
----- FLASH kinetics
_____ Conventional kinetics
